# Chromatographic Phospholipid Trapping for Automated H/D Exchange Mass Spectrometry of Membrane Protein–Lipid Assemblies

**DOI:** 10.1021/acs.analchem.2c04876

**Published:** 2023-01-27

**Authors:** Dietmar Hammerschmid, Valeria Calvaresi, Chloe Bailey, Benjamin Russell Lewis, Argyris Politis, Michael Morris, Laetitia Denbigh, Malcolm Anderson, Eamonn Reading

**Affiliations:** † Department of Chemistry, King’s College London, 7 Trinity Street, SE1 1DB London, U.K.; ‡ Waters Corporation, Stamford Avenue, Altrincham Road, SK9 4AX Wilmslow, U.K.

## Abstract

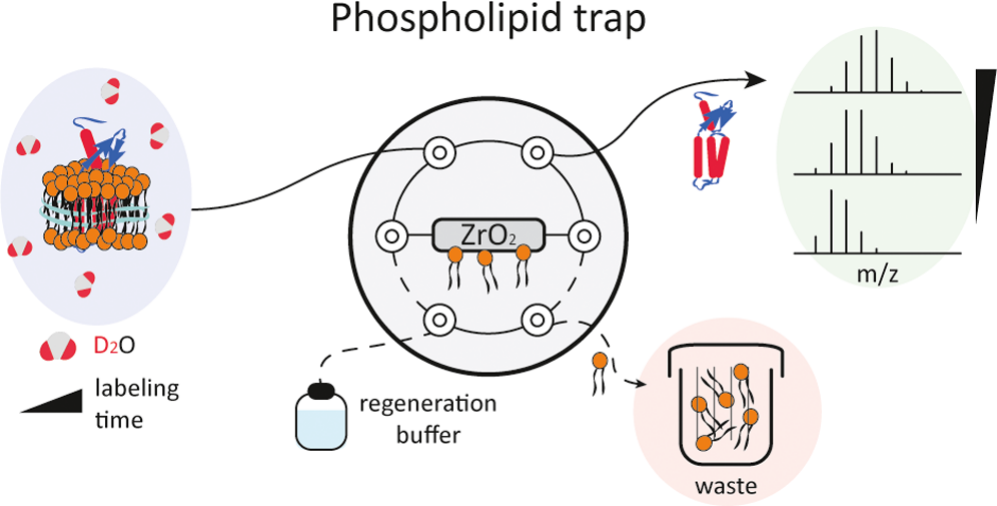

Lipid interactions modulate the function, folding, structure, and organization of membrane proteins. Hydrogen/deuterium exchange mass spectrometry (HDX-MS) has emerged as a useful tool to understand the structural dynamics of these proteins within lipid environments. Lipids, however, have proven problematic for HDX-MS analysis of membrane-embedded proteins due to their presence of impairing proteolytic digestion, causing liquid chromatography column fouling, ion suppression, and/or mass spectral overlap. Herein, we describe the integration of a chromatographic phospholipid trap column into the HDX-MS apparatus to enable online sample delipidation prior to protease digestion of deuterium-labeled protein–lipid assemblies. We demonstrate the utility of this method on membrane scaffold protein–lipid nanodisc—both empty and loaded with the ∼115 kDa transmembrane protein AcrB—proving efficient and automated phospholipid capture with minimal D-to-H back-exchange, peptide carry-over, and protein loss. Our results provide insights into the efficiency of phospholipid capture by ZrO_2_-coated and TiO_2_ beads and describe how solution conditions can be optimized to maximize not only the performance of our online but also the existing offline, delipidation workflows for HDX-MS. We envision that this HDX-MS method will significantly ease membrane protein analysis, allowing to better interrogate their dynamics in artificial lipid bilayers or even native cell membranes.

Membrane proteins have an intimate relationship with their surrounding lipid bilayer.^1−5^ The amphipathic nature of the lipid bilayer combined with the high degree of hydrophobicity possessed by membrane proteins makes their study significantly more difficult compared to their soluble protein counterparts. To interrogate these systems, new analytical tools are required; the importance of this endeavor being intensified by the fact that membrane proteins are key targets for more than half of modern drugs.^6^

Protocols have been established to enable hydrogen/deuterium exchange mass spectrometry (HDX-MS) analysis of membrane proteins within lipid vesicles,^7^ liposomes,^8^ nanodiscs,^9−12^ and so-called “native nanodiscs,” which allow membrane proteins to stay in contact with the native lipid milieu.^5,13,14^ In HDX-MS, a protein is diluted into a deuterated buffer enabling H/D exchange of its labile backbone amide hydrogens. This reaction is quenched at different time intervals by dropping pH and temperature to 2.5 and 0 °C, respectively. The quenched protein sample is then digested by an acid-labile protease (e.g., pepsin) into peptides and the incorporation of deuterium measured by liquid chromatography (LC)-MS analysis. Post deuterium labeling, however, lipids can cause manifold issues in the bottom-up HDX-MS workflow.^15^ These problems range from a reduced protein digestion efficiency, due to potential interference with the protease, to fouling of the liquid chromatography system, peptide–lipid co-elution that adds to spectral complexity, and peptide ion suppression.

The addition of ZrO_2_-coated beads post deuterium labeling offers a sophisticated strategy for depriving the protein samples of lipid components under HDX quench conditions.^16^ Yet, beads need to be removed through filtration before sample injection into a mass spectrometer, which is laborious and time-consuming and thus may affect reproducibility among technical replicates and inevitably increase the D-to-H back-exchange.^17−20^ This process has recently been automated for HDX-MS applications. Anderson et al.^21^ developed a robot-assisted workflow with nanofilter vials, where the labeled protein is transferred to the base of a filter system containing ZrO_2_ beads, and after binding of phospholipids, the sample is filtered through a nanofilter cartridge by a LEAP X-Press module harvesting the protein for subsequent injection. The ZrO_2_ beads and cartridge are then disposed after each experiment. Other approaches for phospholipid removal that avoid the use of ZrO_2_ beads have also been developed, including TCA precipitation^7^ and the use of size exclusion chromatography (SEC).^22^ Herein, we report an alternative chromatographic approach, which enables online, regeneratable phospholipid trapping by a ZrO_2_ bead column. This exploits the Lewis acid chemistry of ZrO_2_—while avoiding the need for precipitation, size separation, filtration, or bead disposal—to offer a robust automated system for HDX, digestion, and LC-MS analysis of proteins within lipid environments.

We determined crucial parameters, such as delipidation efficiency, system robustness, and back-exchange levels, and minimized unspecific protein/peptide adsorption to the column matrix. We also investigated titanium oxide (TiO_2_) beads as an alternative to ZrO_2_-based protein delipidation. Furthermore, we established an automated wash method for bead regeneration in parallel to peptide LC-MS analysis, which enables intervention-free sample acquisition and makes the phospholipid trap column reusable. Finally, we demonstrated the functionality of the delipidation protocol by HDX-MS analysis of empty and loaded membrane scaffold protein phospholipid nanodiscs, and the latter loaded examples containing multidrug efflux pump transmembrane subunit AcrB. We envision that this automated and robust delipidation workflow will make HDX-MS analysis of membrane-embedded proteins routine.

## Experimental Section

### Instrumentation (Standard HDX)

All experiments were performed on an ultraperformance liquid chromatography (UPLC) system (nanoACQUITY, Waters, Wilmslow, UK) coupled to an electrospray ionization quadrupole time-of-flight (ESI-Q-ToF) mass spectrometer (Xevo G2-XS, Waters, Wilmslow, UK). The standard nanoACQUITY system contains a refrigerated HDX manager with a two-valve configuration, i.e., injection and trapping valves, and supplies solvent flow via auxiliary (ASM) and binary (BSM) solvent managers. The HDX manager was kept at 0 °C during all measurements.

### Delipidation Setup (Extended HDX)

**Figure 1:**
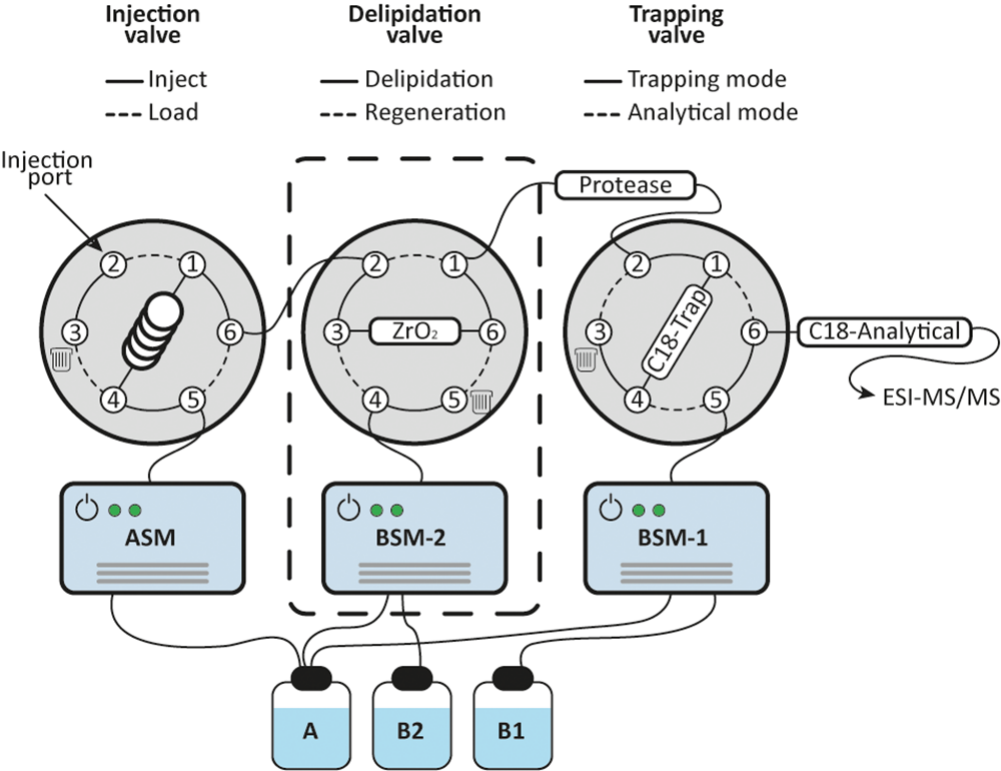
Schematic illustration of the automated phospholipid trapping workflow. The conventional two-valve configuration in standard HDX-MS is extended by an additional valve (dashed box) flanked by injection and trapping valves. The delipidation valve is equipped with a phospholipid trap column and operated by an extra BSM to provide independence from standard HDX-MS solvents. In this configuration, the sample passes through the phospholipid trap column, where lipids are retained, and the protein is transported further to the protease column, following the standard bottom-up workflow of protein digestion, peptide trapping, and subsequent analysis. After delipidation and protein digestion, the ZrO_2_ column can be cleaned simultaneously for peptide analysis using BSM-2.

The standard configuration was extended by an additional “delipidation” valve, which was positioned between injection and trapping valves (Figure 1). The delipidation valve was equipped with an in-house packed phospholipid trap column (ZrO_2_ or TiO_2_), which was kept on ice (Figure S1C).

### Column Packing

Columns for a chromatographic phospholipid trap column (ZrO_2_, Cat No. 5425-U, Supelco, or TiO_2_, Cat No. GL-5020-75000, GL Sciences) and protein digestion (pepsin agarose resin, Cat No. 20343; Thermo Fisher) were packed in-house using both a Microbore Guard Column (1.0 mm ID x 2 cm unpacked; Part No. C-128) and an Analytical Guard Column (2.0 mm ID x 2 cm unpacked; Part No. C-130B) from UVISION Technologies (London, UK). Beads were resuspended and washed in solvent A (0.23% formic acid in H_2_O, pH 2.5). Column parts were cleaned by sonication in solvent A. The column was assembled without the frit (Figure S1B) on the side from which the column was packed using a syringe with an appropriate adapter. After packing, the missing frit was inserted, and the column was flushed back-to-back with solvent A by applying constant pressure with the ASM for a couple of minutes, allowing the bead matrix to settle.

### BSA Blocking of ZrO_2_ Beads

A 3% BSA solution in solvent A was prepared for blocking unspecific sites of ZrO_2_ beads. Beads were washed once in solvent A. The 3% BSA solution was added to the beads, and beads were incubated under gentle agitation for 1 h. Excess of BSA was removed by washing the beads three more times in solvent A before packing into a column (see Column Packing section).

### Lipid Measurements

The HDX manager was equipped with a Vanguard column (BEH C4, 300 Å, 1.7 μm, 2.1 mm × 5 mm; Waters) only. Lipids were trapped on the C4 column and washed with solvent A for 3 min at 200 μL/min. Subsequently, lipids were eluted by a 3 min linear gradient from 8 to 95% solvent B (0.23% formic acid in acetonitrile, pH 2.5) at 40 μL/min. Eluted lipids were measured in positive ion mode between 50 and 2000 m/z on the Xevo G2-XS mass spectrometer. The phospholipid trap column was cleaned with 3% NH_4_OH in methanol and re-equilibrated in solvent A during the subsequent wash run. Experiments were performed in the standard two-valve and extended three-valve HDX-MS configuration. Detailed LC settings are provided in the Supporting Information (Tables S1 and S2). The extracted ion chromatogram (EIC) of the respective lipid was generated, and mass spectra were combined at full width half-maximum (FWHM). The obtained intensity read was used to calculate the delipidation efficiency of the column/system.

### Protein Measurements

PhosB was solubilized in equilibration buffer (10 mM potassium phosphate, pH 7.0) and diluted 1:1 (vol/vol) with quench buffer (100 mM potassium phosphate, pH 2.3). The HDX manager of the nanoACQUITY system was equipped with a Vanguard column (BEH C18, 130 Å, 1.7 μm, 2.1 mm × 5 mm; Waters) and an Acquity UPLC column (BEH C18, 130 Å, 1.7 μm, 1.0 mm × 100 mm; Waters) for peptide trapping and separation, respectively. Protein digestion was performed online with the UPLC chromatographic system using an in-house packed protease column (immobilized pepsin agarose resin) at 15 °C. The generated peptides were trapped and washed with solvent A at 200 μL/min for 3 min. Subsequently, peptides were separated by applying a 7.5 min linear gradient from 8 to 35% solvent B at 40 μL/min. Peptides were measured in positive ion mode between 50 and 2000 m/z on the Xevo G2-XS mass spectrometer. Experiments were performed in triplicate on the standard two-valve and extended three-valve configurations applying a standard bottom-up HDX-MS workflow. Detailed LC settings are provided in the Supporting Information (Tables S3 and S5).

### Evaluation of Back-Exchange

PhosB was digested on the in-house packed protease column at a flow rate of 200 μL/min. The generated peptides were collected for 1 min and subsequently freeze-dried for 5 h. Peptides were resuspended in deuterated labeling buffer (10 mM potassium phosphate, pH_read_ 6.6; 100% final D_2_O content) for 4 h. The reaction was quenched by adding 1:1 (vol/vol) ice-cold quench buffer (500 mM glycine-HCl, pH 2.35). Measurements were performed in triplicate on both standard two-valve and extended three-valve configurations. Peptide trapping and separation was performed on standard C18 trap and analytical columns at 0 °C. The protease column was replaced by a union, and the compartment was kept at 15 °C. The phospholipid trap column was kept on ice. Detailed LC settings are provided in the Supporting Information (Tables S3 and S5).

### Evaluation of Peptide Carry-Over

PhosB was solubilized in equilibration buffer (10 mM potassium phosphate, pH 7.0) and diluted 1:1 (vol/vol) by the addition of ice-cold quench buffer (500 mM glycine-HCl, pH 2.35). Measurements were performed in three subsequent runs with standard wash runs, i.e., pepsin wash (1.6 M guanidinium-HCl, 4% acetonitrile, 0.8% formic acid), in between. Then, a blank run (1:1 mix of equilibration and quench buffer) was performed, and carry-over was evaluated based on peptide intensities. Detailed LC settings are provided in the Supporting Information (Tables S3 and S5).

### H/D Exchange Mass Spectrometry of Empty MSP1E3D1 Nanodiscs

The extended HDX valve configuration was used and equipped with a ZrO_2_-packed phospholipid trap column (kept on ice) upstream an in-house packed pepsin column (kept at 15 °C). The HDX manager was equipped with a Vanguard column (BEH C18, 130 Å, 1.7 μm, 2.1 mm × 5 mm; Waters) and an Acquity UPLC column (BEH C18, 130 Å, 1.7 μm, 1.0 mm × 100 mm; Waters) for peptide trapping and separation, respectively. Deuterium labeling was performed with a PAL3 RTC HDX robot (Trajan Scientific, Morrisville). MSP1E3D1 nanodiscs, both POPC (1:85 protein/lipid) and EPL (1:60 protein/lipid), were diluted 20-fold (95% D_2_O final) into deuterated labeling buffer (20 mM Tris, 100 mM NaCl, 0.5 mM EDTA, pH_read_ 7.0) for 10, 100, 1000, and 10,000 s at 20 °C. References were performed in nondeuterated equilibration buffer. The reaction was quenched by adding 1:1 (vol/vol) ice-cold nanodisc quench buffer (500 mM glycine-HCl, 1.6 M guanidinium-HCl, 0.8 mM Na-cholate, pH 2.35). Three technical replicates were performed with a standard bottom-up HDX-MS workflow applying a 7.5 min linear gradient from 8 to 35% solvent B at 40 μL/min. Peptides were measured in positive ion mode between 50 and 2000 m/z on the Xevo G2-XS mass spectrometer, applying settings to minimize gas-phase back-exchange.^23^ The phospholipid trap column was cleaned with 3% NH_4_OH in methanol and re-equilibrated in solvent A during the subsequent wash run. Labeling experiments were also performed on free-soluble MSP1E3D1 (95% D_2_O for 10, 100, 1000, and 10,000 s), which was measured on both standard two- and extended three-valve configurations. Detailed LC settings are provided in the Supporting Information (Tables S3 and S5).

### Mass Spectrometry of AcrB Nanodiscs

AcrB nanodiscs were equilibrated in nondeuterated AcrB sample buffer (no D_2_O labeling performed), and ice-cold nanodisc quench buffer was added 1:1 (vol/vol). Three technical replicates were performed with a standard bottom-up HDX-MS workflow applying a 9.0 min linear gradient from 8 to 40% solvent B at 40 μL/min (Figure S4). The total protein amount injected was 20 pmol. Detailed LC settings are provided in the Supporting Information (Tables S4 and S5).

### Data Processing

Lipid spectra were processed with MassLynx 4.2 (Waters, Wilmslow, UK). Protein identification (PhosB and MSP1E3D1) and peptide filtering were performed with ProteinLynx Global Server 3.0 (PLGS) and DynamX 3.0, respectively (Waters, Wilmslow, UK). PLGS workflow parameters for peptide identification were as follows: peptide tolerance: automatic; fragment tolerance: automatic; min fragment ion matches per peptide: 2; minimum fragment ion matches per protein: 7; minimum peptide matches per protein: 3; maximum protein mass 250,000; primary digest reagent: nonspecific; and false discovery rate: 100. DynamX parameters for peptide filtering were as follows: minimum intensity: 1481; minimum sequence length: 5; maximum sequence length: 25; minimum products per amino acid: 0.11; minimum consecutive products: 1; minimum score: 6.62; maximum MH+ error (ppm): 5; file threshold: n-1.^24^ Bimodal isotopic envelope analysis was performed with HX-Express2 on the MSP1E3D1 peptide WDNLEKETEGLRQEMSKD after spectra were smoothed 4 × 2 using Savitzky–Golay in MassLynx.^25^

## Results and Discussion

### Automated Phospholipid Trapping

Automation endeavors always aim for both repeatability—ideally free of user interventions—and system robustness. To meet these requirements for the automated trapping of phospholipids in HDX-MS experiments, we integrated an additional valve online with the chromatographic system but placed it outside the standard two-valve HDX chamber, conventionally used for standard bottom-up HDX-MS analysis (Figure 1). The delipidation valve is equipped with a phospholipid trap column, which is kept refrigerated in a polystyrene box containing ice (0 °C) and supplied with eluents by an additional binary solvent manager (BSM) to provide independence from standard HDX-MS solvents. The integration of the additional valve and phospholipid trap column leads to an increase in pressure of ∼1000 psi (∼4000 psi final) during the sample load, which originated from the valve itself and has no unbeneficial effect on the performance of both phospholipid trap and protease column. The three-valve system is of straightforward use and fully automated (in our case, controlled by Waters MassLynx software) and can be coupled to a robot performing automated deuterium labeling and sample injection.

The developed online delipidation method—with integrated phospholipid trap—works as follows. (1) After the sample injection, the sample components are fed by an auxiliary solvent manager (ASM) through the phospholipid trap column, where lipids are retained through their interaction with ZrO_2_ or TiO_2_ while the protein passes through. (2) The protein is further guided to the online protease column for digestion, and generated peptides are captured in the peptide trap column for desalting. (3) At the end of the trapping time, the phospholipid trap column is automatically configured offline with the protease column. While the latter can be selectively flushed with a protease wash solution, the phospholipid trap column is washed with an alternate solvent (which we denote as solvent B2) for removal of retained phospholipids, which are directed to a waste compartment. The cleaning step (regeneration) of the phospholipid trap column occurs simultaneously with LC peptide separation, thus with no extra time added to the sample run. (4) In conventional HDX-MS measurements, following each protein sample run, the analytical segments are usually washed with a sawtooth-gradient run. In the three-valve system, during this wash run, the phospholipid trap column remains configured offline from the protease column and is re-equilibrated with solvent A (typically 0.23% formic acid), preparing it for the subsequent sample injection. The three-valve configuration also provides flexibility, as the phospholipid trap column can be positioned up- or downstream the protease column, allowing sample delipidation to be performed at protein or peptide level, respectively, without requiring further modifications on the LC methods. A potential downside of this approach, however, would be that phosphopeptides are also likely to be depleted while passing through the phospholipid trap column, although whether phosphorylated proteins would be trapped remains untested.

### Determining the Delipidation Efficiency

To investigate whether phospholipids are retained after passing through the phospholipid trap column, we measured lipids on both the standard two-valve and the extended three-valve HDX-MS system. Initially, we injected four 1-palmitoyl-2-oleoyl-sn-glycero-3-phosphocholine (POPC) aggregates (0.01, 0.1, 1, and 10 pmol) in duplicate and plotted the obtained MS signal against the amount of lipid (Figure S5A). After that, we increased the concentration of POPC and ran triplicate over the delipidation system (three-valve configuration), applying both the ZrO_2_ and TiO_2_ columns. The remaining POPC signal after phospholipid trapping was used to calculate the delipidation efficiencies for the applied column/system.

**Figure 2:**
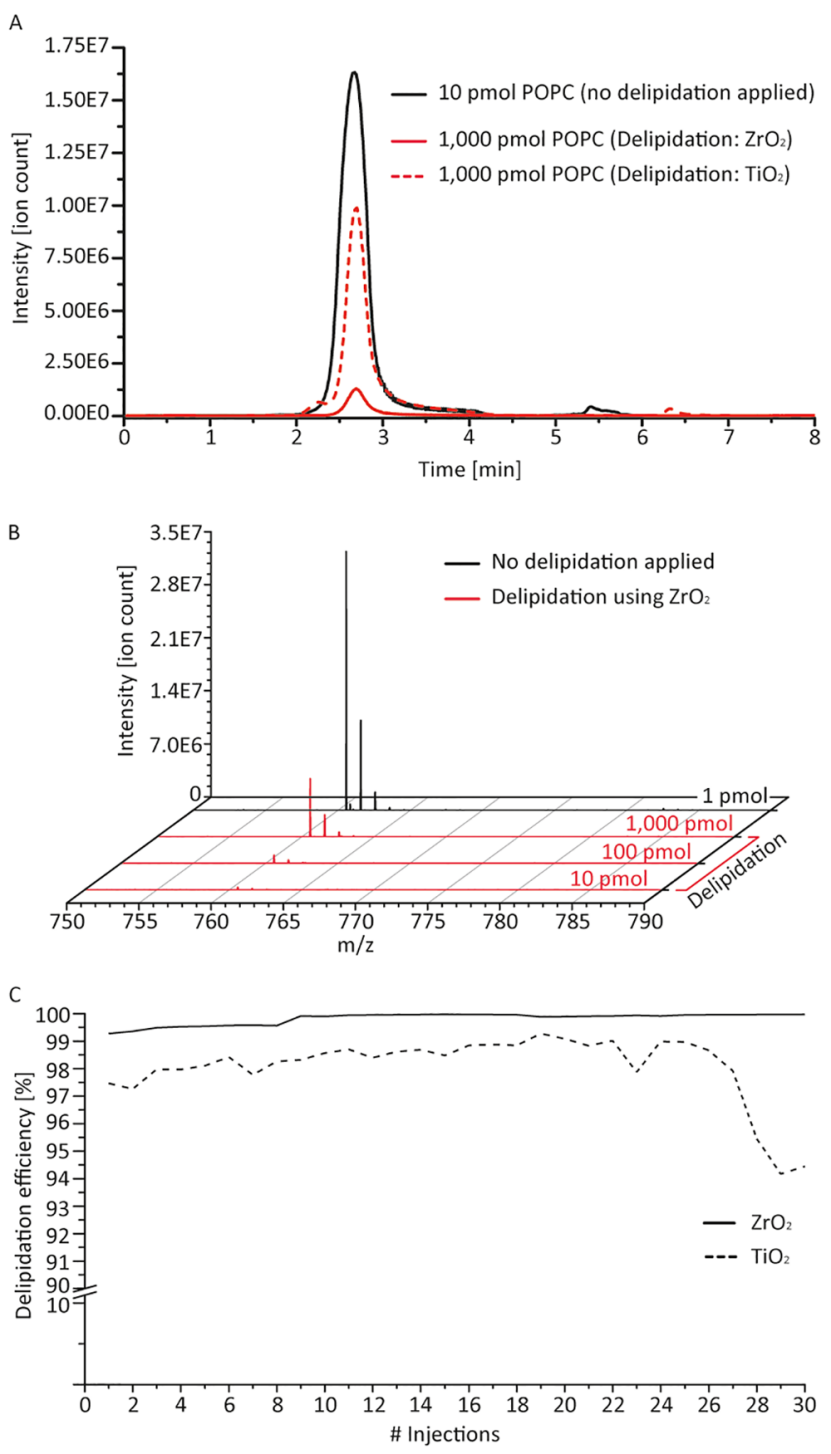
Delipidation efficiency and system robustness of the automated phospholipid trapping workflow. (A) Extracted ion chromatogram of POPC (760.6 m/z) before (solid black) and after (ZrO_2_: solid red, TiO_2_: dashed red) applying online sample delipidation. (B) Combined mass spectra (scan 280:330) of various POPC amounts acquired with (solid red) and without (solid black) the ZrO_2_ trap column in place. (C) Delipidation efficiency of both bead types (ZrO_2_: solid line, TiO_2_: dashed line) over the course of 30 POPC injections with appropriate column cleaning in between. Delipidation measurements have been performed on unblocked ZrO_2_ or TiO_2_ columns.

The extracted ion chromatogram (EIC) as well as the MS signal of POPC impressively illustrates a >100–1000-fold lipid reduction for both bead types, with ZrO_2_ outperforming TiO_2_ (Figure 2A,B). We performed similar lipid trapping experiments applying an *Escherichia coli* lipid extract (EPL) and Fos-choline-12. Again, we plotted the intensity of the lipid signal against the injected amount (Figure S5B,C) and exploited the remaining MS signal to calculate the system’s delipidation capacity (Tables 1, S6, S7; Figure S6). For EPL, we determined lipid removal efficiency for each lipid individually, which is ∼96% for ZrO_2_ and ∼87% for TiO_2_, slightly lower than for POPC (Tables 1, S6, S7). Both bead types, however, do not show any discrimination in terms of lipid length (Figure S7).

**Table 1:** Delipidation Capacity of ZrO_2_ and TiO_2_^a^

			efficiency [%]	
lipid/sample	column	lipid amount	ZrO_2_	TiO_2_
Unblocked Column (No Blocking of Unspecific Sites with BSA)				
POPC	2 × 20 mm	10 pmol	99.97 ± 0.01	99.96 ± 0.01
POPC in DDM	2 × 20 mm	10 pmol	31.94 ± 6.92	60.26 ± 1.65
POPC in Fos-choline-12	2 × 20 mm	10 pmol	99.83 ± 0.06	99.71 ± 0.04
EPL (PE 33:1)	2 × 20 mm	10 ng	97.30 ± 0.33	86.90 ± 0.50
Fos-choline-12	2 × 20 mm	100/10 pmol	99.99 ± 0.00	7.39 ± 4.38
POPC	no column (offline)	10 pmol	95.44 ± 0.21	ND
POPC	1 × 20 mm	10 pmol	98.95 ± 0.29	ND
BSA Blocked Column				
POPC	1 × 20 mm	10 pmol	89.03 ± 0.49	ND
POPC (optimized quench buffer)	1 × 20 mm	10 pmol	90.59 ± 1.00	ND

a Overview of delipidation efficiencies of ZrO_2_ and TiO_2_ beads determined for different column dimensions and a variety of phospholipids with and without the presence of detergents. A detailed overview of all delipidation rates is provided in Tables S6–S8. ND, not determined.

In standard HDX-MS analysis of membrane proteins, detergents are usually added to the quench buffer to facilitate nanodisc rupture.^10,13^ Hence, we also determined the delipidation capacity in the presence of detergents, e.g., 0.1% DDM or 0.1% Fos-choline-12 (a concentration above their critical micelle concentration). DDM causes a significant drop in delipidation efficiency (32 and 60% of POPC removal for ZrO_2_ and TiO_2_, respectively, Table 1), potentially due to steric hindrance through a bigger micelle formation. However, Fos-choline-12 exerts no detrimental effect in terms of measured lipid removal. Moreover, despite structural similarities with phospholipids, i.e., the phosphatidylcholine headgroup, TiO_2_ fails to retain Fos-choline-12, while ZrO_2_ shows an even stronger binding than for POPC (Table 1; Figure S6). Therefore, while the presence of DDM in the quench buffer appears disadvantageous, Fos-choline-12 appears highly suitable as a delipidation-compatible quench buffer additive, as ZrO_2_ beads also prevent the disadvantageous Fos-choline-12 contamination of the downstream chromatography and MS source. Current workflows perform the delipidation step offline from the UPLC system,^10,13,21^ which greatly differs from the online chromatographic approach presented here. Comparing both approaches in terms of their lipid removal capacity reveals a better performance of the column-based workflow introduced here (Table 1), adding another advantage to the automation benefit.

### Column Regeneration

Recurring lipid injections requires the phospholipid trap column to be cleaned to retain delipidation capacity over a long period of time. We tested the suitability of different MS-compatible solvents, such as acetonitrile, isopropanol, and methanol, for cleaning the phospholipid trap column. Standard protocols in phosphoproteomics apply an increasing basicity with ammonium hydroxide (NH_4_OH) to elute phosphopeptides from TiO_2_,^26−30^ providing an alternative to organic solvents. To investigate the lipid cleaning capacity of the different solvents, we loaded 1 pmol of POPC on the ZrO_2_ column and subsequently applied a sawtooth gradient of 5 × 2 min washes from 0–100% solvent B2 (Figure S8). Success, i.e., cleaning of the phospholipid trap column, would not only be indicated by MS detection of the lipid but also by decreasing its signal intensity over various gradient cycles. Such an EIC profile is displayed for methanol and 3% NH_4_OH, which performs even better in combination (Figure S8).

### Delipidation System Robustness

We investigated the robustness of the entire delipidation system by recurring POPC injections (30 injections) with appropriate cleaning (3% NH4OH in methanol) of the phospholipid trap column, both ZrO_2_ and TiO_2_, in between. Advantageously, the column regeneration occurs simultaneously to the analytical gradient. The subsequent wash run—a step commonly applied to prevent sample carry-over—was applied to not only clean the C4 trap column but also to re-equilibrate the phospholipid trap column to solvent A (0.23% formic acid), i.e., to pH 2.5. The delipidation efficiency is comparable with previous results (Table 1) and, most impressively, remains over 99% for ZrO_2_ throughout (Figure 2C). The delipidation rates obtained for TiO_2_ are slightly lower (∼97–98%), yet reasonably sufficient, and show a drop of 3% within the last three injections.

### ZrO_2_ or TiO_2_ Trap/Protein Interactions

**Figure 3:**
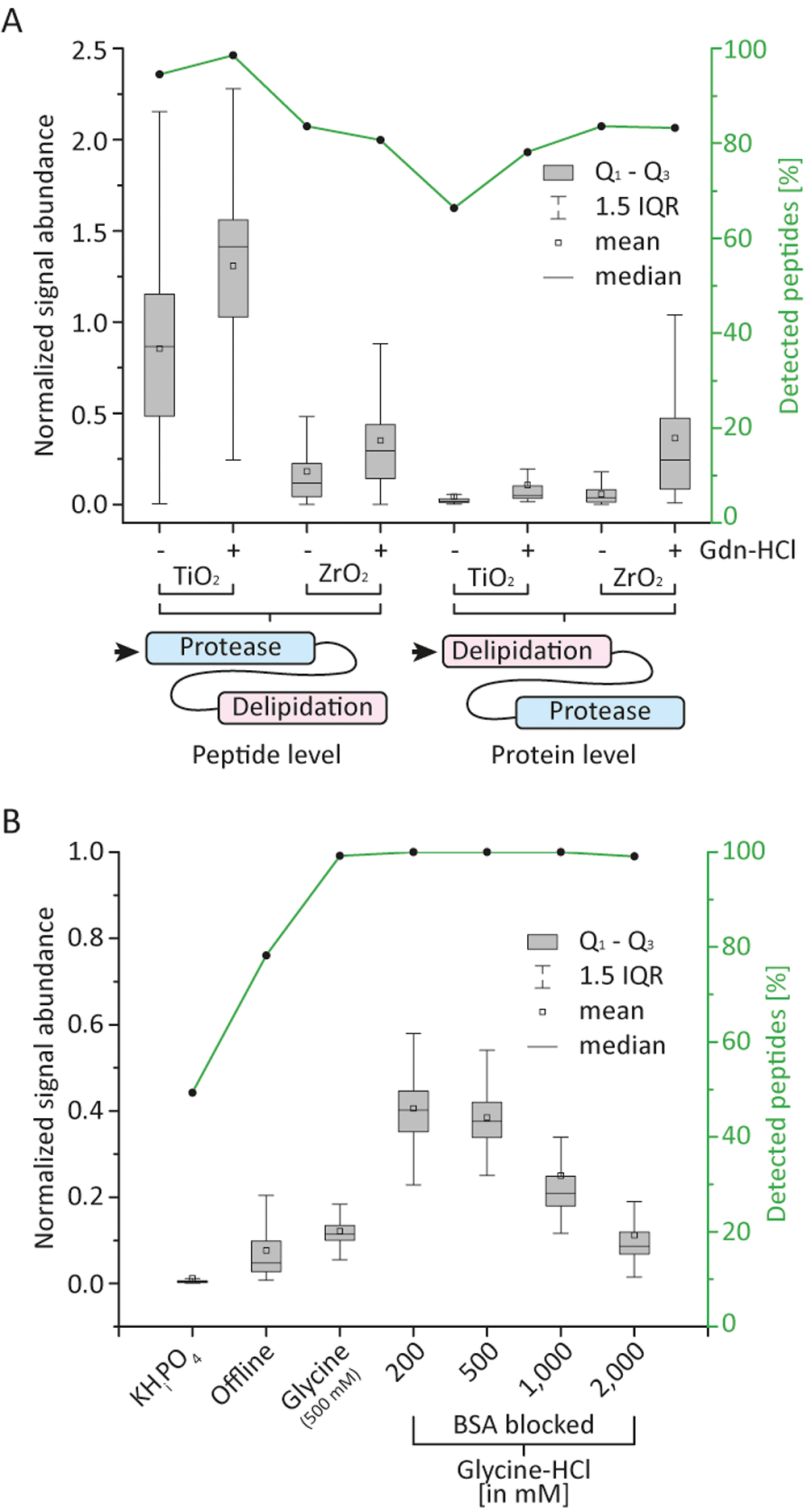
Unspecific adsorption of proteins/peptides to the stationary phase of the phospholipid trap column. (A) Boxplot representing the normalized peptide signal abundance of measurements performed on the delipidation system relative compared to the standard two-valve HDX-MS configuration. Experiments were conducted on both protein and peptide levels ± 1.5 M guanidinium-HCl using either a TiO_2_- or a ZrO_2_-based column. (B) Normalized peptide intensities after applying different glycine-HCl quench buffers with and without BSA bead blocking in comparison with phosphate buffer (protein level only). Experiments with phosphate buffer were performed either automated (column-based) or manual after the addition of ZrO_2_ beads with subsequent filtering of the sample. The *y*-axis on the right-hand side (green) indicates the percentage of peptides with sufficiently high signal-to-noise ratios for reliable peak assignment. IQR, interquartile range. Both glycine-HCl and potassium phosphate (100 mM) quench buffers were mixed 1:1 (vol/vol) with PhosB in 10 mM potassium phosphate, pH 7.0.

The integration of an additional column into the delipidation system might lead to unfavorable, unspecific interactions between the target protein and the phospholipid trap column matrix. To investigate such unspecific adsorption effects, we conducted bottom-up PhosB measurements on both systems, i.e., standard two-valve and extended three-valve HDX-MS configurations. We solubilized PhosB in 10 mM potassium phosphate (pH 7.0) and diluted it in 100 mM potassium phosphate (pH 2.3), simulating HDX quench conditions (final pH of 2.5 at 0 °C). Then, we compared the peptide intensities obtained before and after passing through the TiO_2_ or ZrO_2_ column. We measured the extent of unspecific binding of PhoB to the phospholipid trap column on both protein and peptide level, as the phospholipid trap column can be operated up- and downstream the protease column. Unfortunately, the degree of unspecific column/peptide and, in particular, column/protein interactions was significant (Figure 3A), making reliable peptide measurements difficult not to say impossible. Put simply, unspecific adsorption effects are more severe for intact protein than on the peptide level. Only TiO_2_/peptide interactions seem to be mostly negligible. On the protein level, the loss in intensity amounts to two orders of magnitude on average independent of the bead type, which decreases the signal-to-noise ratio to an unacceptable degree for most peptides. We also calculated the percentage of detected peptides compared to control measurements performed in the two-valve configuration. The loss of identifiable peptides ranges from 15 to 30% on the protein level for both bead types and for the ZrO_2_/peptide configuration (Figure 3A). Solely the setup for TiO_2_/peptide measurements is acceptable in terms of peptide loss. The addition of a chaotropic agent to the quench buffer, e.g., 3.0 M guanidinium-HCl (1.5 M final) as a potential suppressor of unspecific protein/peptide adsorption leads to a marginal increase of signal. The number of undetected peptides, however, remains largely unimproved, accounting still for 15–20%.

Following this, we intensified our endeavors to prevent unspecific adsorption to the column matrix. For this purpose, we (i) cut the column volume by 3/4, i.e., halving the column diameter to 1 mm, and (ii) tested different quench buffers to potentially shield unspecific binding sites. At this point, we also decided to solely focus on ZrO_2_ beads as TiO_2_ shows (i) a lower delipidation capacity and (ii) no benefit in terms of unspecific binding on the protein level**—**the preferred configuration, as lipids are ideally removed pre-digestion to not hamper proteolysis.^31,32^ The smaller phospholipid trap column performs equally efficiently (∼99%) in sample delipidation as demonstrated for the bigger column (Table 1).

We applied the following quench buffers: (i) solvent A (0.23% formic acid), (ii) 500 mM glycine-HCl pH 2.35, (iii) 5 mg/mL 2,5-dihydroxybenzoic acid (DHB) in H_2_O, (iv) 15 mg/mL DL-lactic acid in H_2_O and compared them with the standard quench (100 mM potassium phosphate, pH 2.3) (Figure S9). All quench buffers led to a pH of 2.5 upon 1:1 mix (vol/vol) with the standard protein buffer (10 mM potassium phosphate pH 7.0).

DHB and DL-lactic acid were selected as they have previously proven beneficial to prevent unspecific binding in phosphoproteomics.^30,33^ Glycine-HCl was chosen for two reasons. First, it is already known as a reliable quench buffer in HDX-MS.^34−36^ Second, amino acids, e.g., arginine, have shown to potentially prevent unspecific protein binding in size exclusion chromatography.^37,38^

DL-lactic acid exhibits minor but unsatisfactory improvements, while other quench buffers, i.e., solvent A (0.23% formic acid) and DHB, do not lead to any performance gain. The quench buffer of 500 mM glycine-HCl appears to prevent unspecific interaction sites in the ZrO_2_ matrix. The average loss of peptide intensity is roughly one order of magnitude (90%) still high, yet, more importantly, almost 100% of the peptides could be recovered through a sufficient signal-to-noise ratio (Figure S9). Quite unexpectedly though, with an increasing number of technical replicates performed, we observed that the issue of unspecific interactions between protein and ZrO_2_ beads became less severe, as evidenced by the obtained chromatograms (Figure S10). This phenomenon could be explained by the protein increasingly “blocking” unspecific binding sites of the beads. To test whether unspecific protein adsorption could be reduced by protein bead blocking, we prepared a phospholipid ZrO_2_ bead trap column blocked with 3% bovine serum albumin (BSA) solubilized in solvent A (0.23% formic acid). BSA is not only a common blocking agent in immunoassays to prevent unspecific protein binding,^39^ but has also been applied in combination with ZrO_2_ beads to obtain robust and reliable results in cell lysis assays.^40^ We then injected PhosB over the blocked and unblocked ZrO_2_ phospholipid trap columns using 500 mM glycine-HCl (pH 2.35) as quench buffer. The average peptide signal intensity increases four to five times when ZrO_2_ beads are blocked with BSA (Figure S9).

### Optimized Phospholipid Trap Conditions

Finally, we determined the ideal glycine-HCl concentration in the quench buffer. The best performances, measured in terms of peptide signal intensity and identifications, are obtained with 200 and 500 mM glycine-HCl quench buffers, with no significant differences between the two concentrations. To note, the average peptide signal drops at higher concentrated glycine-HCl quench buffers, which might be explained by peptide ion suppression due to incomplete desalting or a detrimental effect on protein digestion (Figure 3B). We then compared the performance of our optimized online delipidation system, in terms of protein recovery, to the offline ZrO_2_-based delipidation protocol proposed in the literature.^10^ Although a higher amount (25 mg) of beads is used to pack the phospholipid trap column in the online system compared to the 10 μL (3 mg) used in the offline workflow, in our hands, unspecific adsorption in the offline protocol was much higher compared to the automated workflow with glycine-HCl and/or the blockage of the beads (Figure 3B). This experiment highlights that this problem requires to be addressed when beads are handled manually (offline), as reliable measurements are hardly feasible without an adequate strategy to overcome unspecific protein binding. We envision that the BSA blockage of beads and the use of glycine-HCl as quench buffer will be suitable to prevent protein unspecific adsorption to beads also in the offline workflow.

### Back-Exchange and Carry-Over

Back-exchange and peptide carry-over are crucial parameters to control in HDX-MS, which can be negatively influenced by an extended flow path and/or an additional column matrix. To investigate the impact of the integrated delipidation system, we conducted PhosB measurements on both configurations and compared back-exchange and carry-over on a large ensemble of peptides (Figure S11). For the back-exchange control, we predigested PhosB and maximally deuterated the generated peptides with 100% D_2_O. While back-exchange levels unsurprisingly vary across peptides, hardly any differences are observed when comparing the same peptide between the two systems (Figure S11A). This similitude is also reflected by the calculated average of back-exchange, which accounts for 30.02 ± 0.26% in the standard two-valve and 30.34 ± 0.63% in the extended three-valve configuration (Table S9). The assessment of peptide carry-over also highlights that the delipidation system performs equally well as compared to the standard HDX-MS system, with a total average of 0.77% (85% of all peptides show less than 1% carry-over) for both systems. We also measured PhosB in the presence of 1000 pmol POPC (20 times excess) to investigate whether lipids affect carry-over of hydrophobic peptides. In summary, all peptides show comparable degrees of persistence in both configurations as well as in the presence of lipids, which excludes the phospholipid trap column as a source of peptide carry-over (Figure S11B and Table S10).

### HDX-MS Analysis of the Nanodisc Membrane Scaffold Protein Using Online Delipidation

After optimization, we aimed to test the automated delipidation workflow on nanodisc samples. Nanodiscs utilize a membrane scaffold protein (MSP)—a derivative of apolipoprotein A-1—capable of encasing an inner lipid core, providing a phospholipid bilayer for membrane protein solubilization.^41,42^ Thus, the MSP protein exists in lipid-free as well as nanodisc form, which makes this type of lipid vesicle an ideal membrane protein system to test the performance of novel HDX-MS workflows.

**Figure 4:**
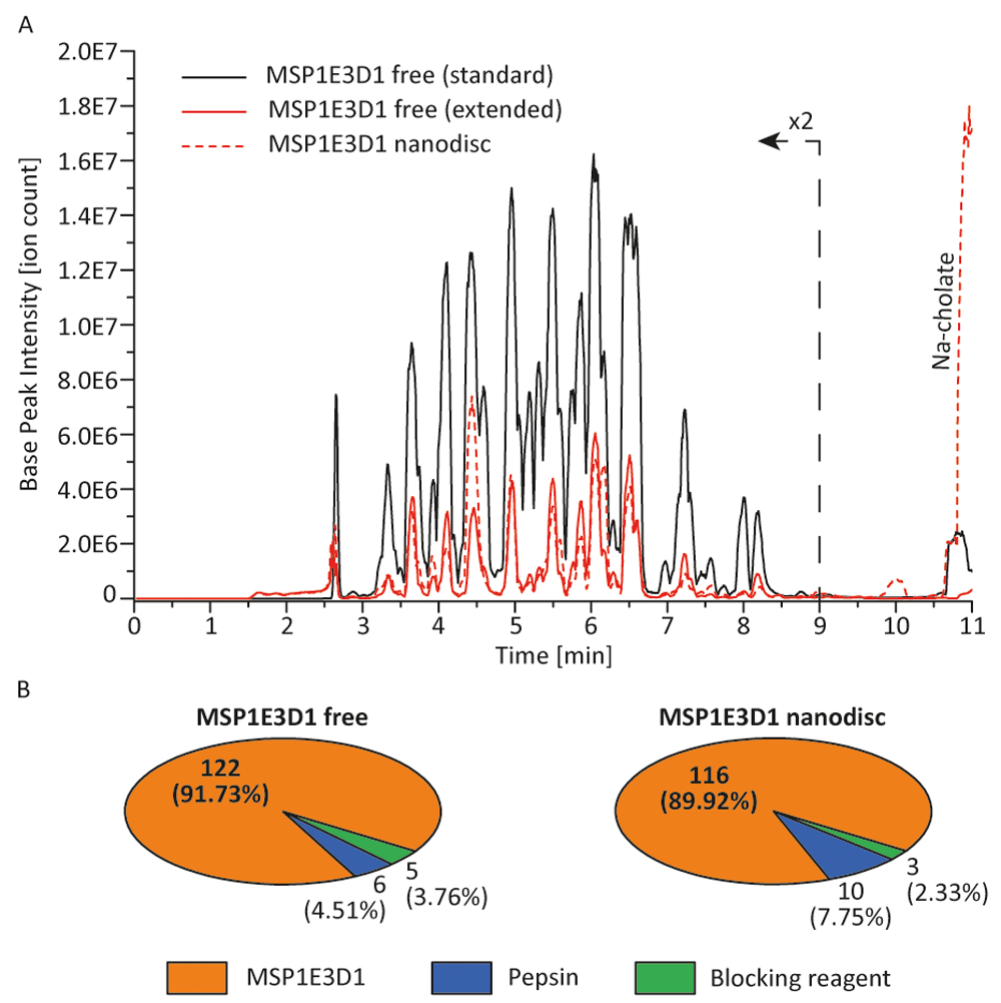
Comparison of MSP1E3D1 (nanodisc) measurements performed on standard two-valve and extended three-valve configurations. (A) Base peak chromatogram of free MSP1E3D1 measured in standard (solid black) and extended (solid red) HDX-MS configuration as well as of nanodisc MSP1E3D1 (dashed red). (B) Number of identified peptides (after filtering with PLGS and DynamX) for the blocking reagent, the protease pepsin, and the MSP1E3D1 protein. Peptides identified for each protein species are also reported as a percentage of total peptides identified.

First, we measured free MSP1E3D1—the utilized MSP protein—on the standard two-valve HDX-MS configuration. Subsequently, we applied the extended delipidation configuration to measure MSP1E3D1 in the free as well as nanodisc state (Figure 4). The base peak ion chromatogram displays 2–3 times lower intensities when performed on the extended three-valve configuration delipidation system (Figure 4A), which is in agreement with previous observations for unspecific protein adsorption under optimized conditions (Figure 3B). More importantly, intensities obtained from analysis on the delipidation system both in the absence and presence of lipids, i.e., free and nanodisc MSP1E3D1, are of equal magnitude. Free MSP1E3D1 yielded a total number of 122 identified peptides in the two-valve configuration compared to 116 peptides obtained for MSP1E3D1 in POPC nanodiscs measured with the three-valve system (Figure 4B). For peptide identification, we not only targeted MSP1E3D1 but also the blocking protein BSA and the primary digestion reagent pepsin. Of interest, the amount of non-MSP1E3D1 peptides accounts for approximately 10% in both experiments, confirming that the phospholipid trap column is only releasing small quantities of the blocking protein. Full sequence coverage could be obtained for MSP1E3D1 in nanodiscs, measured with the three-valve system (Figure S12A)—even slightly better than for free MSP1E3D1 (Figure S12B)—demonstrating the functionality of the automated HDX-MS workflow with online delipidation of nanodisc samples.

As demonstrated, BSA blocking of ZrO_2_ beads was a suitable remedy to overcome crucial unspecific binding of the target protein to the phospholipid trap column. The 2–3 times lower signal intensity observed on the three-valve configuration likely comes from remaining unspecific binding sites in the ZrO_2_-coated silica beads. However, the intensity of the base peak ion chromatogram remains constant throughout the acquisition of all datasets presented (Figure S13), indicating that desorption of BSA is likely minimal not only during analysis but also while the phospholipid trap column is regenerated. Therefore, after initial blocking of unspecific binding sites with BSA, there was no need for re-blocking the phospholipid trap column, at least during a typical HDX-MS experimental run.

Finally, we performed deuterium labeling experiments (ranging from 10 to 10,000 s) on POPC and EPL nanodiscs as well as on free MSP1E3D1. The deuteration of backbone amide hydrogens in native proteins is mediated by transient opening/closing events in their H-bonding networks,^43,44^ which can report on the dynamics of proteins based on their deuterium uptake at different time points.

**Figure 5:**
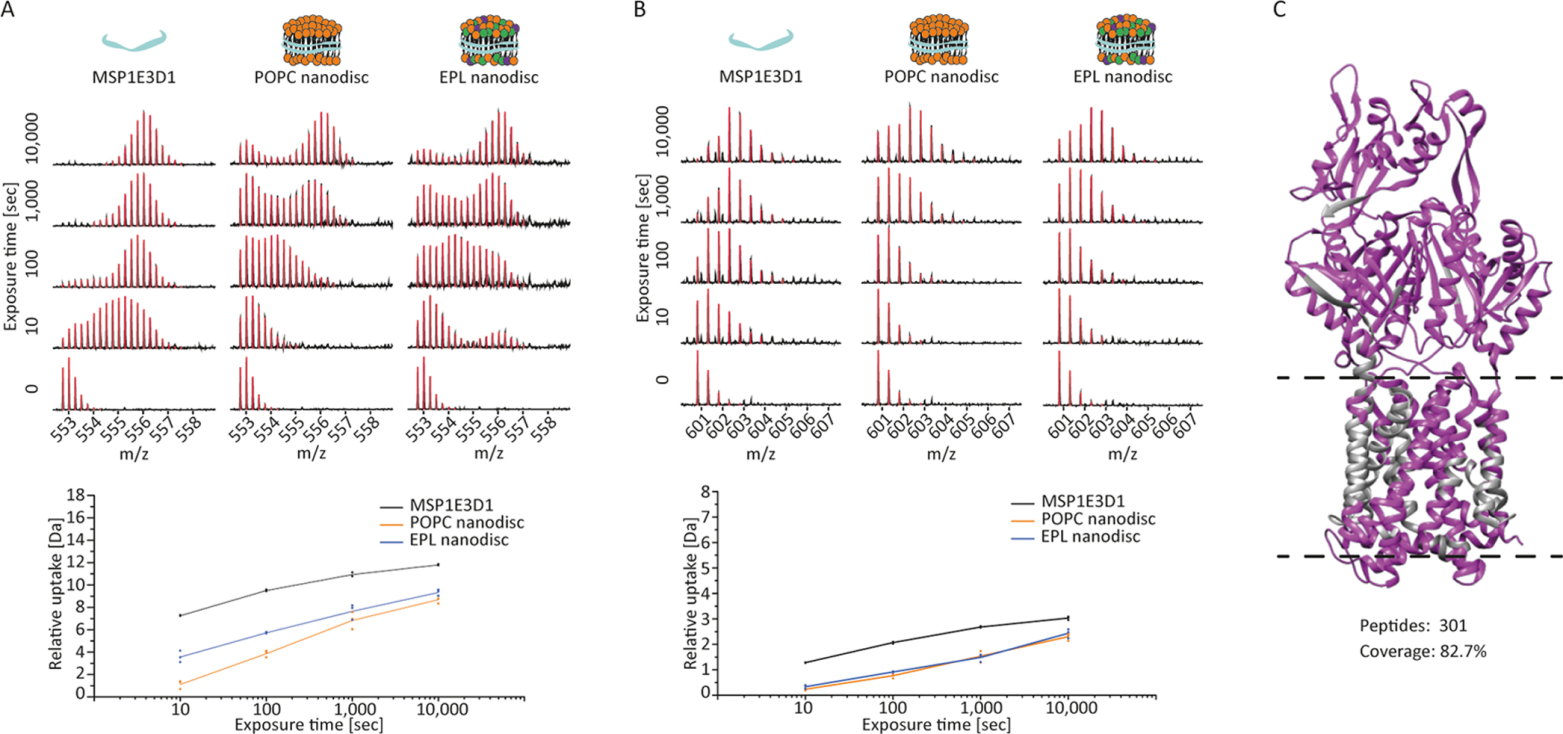
Deuteration of MSP1E3D1 in free, POPC, and EPL nanodisc form. (A) Stacked spectral plot of peptide WDNLEKETEGLRQEMSKD (residues 40–57; m/z 552.76; +4) highlights a bimodal distribution of deuterium uptake. The uptake plot shows a clear difference between both nanodiscs and free MSP1E3D1, indicating a lipid-selective modulation upon disc formation. A detailed analysis of the different spectra (bimodal fitting) is shown in Figure S15. (B) Stacked spectral plot of peptide RTHLAPYLDD (residues 128–137; m/z 600.80; +2). The uptake plot shows again stabilization in both nanodiscs. (C) Sequence coverage of AcrB obtained from measurements in POPC nanodisc (magenta: coverage; gray: no coverage). Dashed lines indicate transmembrane domain.

We observed that MSP1E3D1 predominantly displays a bimodal pattern of deuterium uptake, both in its lipid-free soluble form and when it encases lipids to form nanodiscs (Figure 5A). Importantly, we were able to verify that the isotopic envelope bimodality observed across several peptides of MSP1E3D1 does not result from artifacts, as the three-valve system did not lead to peptide carry-over or abnormal back-exchange (Figure S11). Additionally, bimodality was observed, and at a comparable extent, for the free form analyzed with both the standard two-valve and three-valve (phospholipid trap) systems (Figure S14). The significance and biochemical interpretation of this bimodality warrants further study but likely reflects multiple conformations of MSP1E3D1, which are influenced by their respective lipid environment, nanodisc sizes, and stability.^45^

Importantly, and in line with previous reports,^46^ we found that nanodisc formation leads to stabilization of MSP1E3D1, which is displayed by more intense low-mass envelopes of MSP1E3D1 peptides in both nanodisc forms compared to the soluble form (Figures 5A and S15). Furthermore, peptide spectra show significant differences in the evolution of their deuterium uptake for MSP1E3D1 between the two types of nanodiscs, indicating selective modulations of MSP by nanodisc lipids. Lipid-modulated differences are, however, not always present, as indicated by peptide RTHLAPYLDD (Figure 5B).

Finally, we wanted to test the applicability of the workflow on loaded nanodiscs containing AcrB, an ∼115 kDa transmembrane protein. We measured AcrB solubilized in POPC nanodiscs under optimized quench conditions, and after data processing and peptide filtering, we could obtain 301 peptides and 82.7% sequence coverage (Figure 5C), which is higher than previously reported for AcrB in DDM,^47^ proving the utility of the established online delipidation setup.

## Conclusions

Here, we present an extended HDX-MS system that enables automated sample delipidation for lipid-solubilized membrane proteins. We incorporated a ZrO_2_-packed phospholipid trap column online with protein digestion and subsequent peptide analysis. This setup allows phospholipids to be retained in the ZrO_2_ matrix of the phospholipid trap column, while proteins pass through and undergo digestion with subsequent peptide trapping. Therefore, the system not only provides an automated but also an economic and environmentally friendly way of protein delipidation (i.e., reduction of phospholipid trapping beads and plastic usage). We compared ZrO_2_ and TiO_2_ beads in terms of delipidation efficiency and unspecific protein/peptide binding. ZrO_2_ beads have been shown to outperform TiO_2_ with delipidation efficiency, which was well above 99% for POPC throughout a course of 30 injections. The efficiency of ZrO_2_ beads was also shown to be independent of the type and length of phospholipids, and unlike TiO_2_, even capable of retaining Fos-choline-12. We also assessed the level of back-exchange and peptide carry-over for the extended system, verifying that both do not show any noticeable increase compared to the standard HDX-MS configuration.

The additional column matrix initially led to a significant amount of unspecific protein and peptide binding, which leads to unfavorable loss of peptide signals. Minimizing protein loss was a crucial step and required the optimization of both bead and solution conditions. Increasing the amount of protein injected to obtain a sufficiently high MS signal, a strategy commonly applied so far, is unfavorable for two main reasons: (i) the increase in sample consumption of the target membrane proteins, which are generally obtained in low amounts due to a challenging expression and purification, and (ii) the proportional increase in the amount of lipid components injected at the expense of their effective removal.

We could demonstrate that such protein/bead unspecific adsorption, hence protein loss, can be largely minimized by blocking unspecific binding sites utilizing a combination of blocking reagent, e.g., BSA, and a suitable quench buffer of 200–500 mM glycine-HCl, with only a minor effect on lipid removal efficiencies (Table 1). BSA appeared to remain adsorbed to the ZrO_2_-coated silica beads during sample collection and column regeneration. These observations are supported by computational and experimental studies, which have shown that BSA irreversibly adsorbs to silica—but never fully covers its surface—within acidic, neutral, and basic solution regimes.^48^ BSA blocking is an effective solution to combatting unspecific protein binding during phospholipid removal; however, it could coelute with the protein of interest, complicating the chromatogram and affecting the analysis of the protein of interest. Other protein-free reagents for blocking unspecific protein binding are also available, e.g., SynBlock (Bio-Rad), and might therefore be an attractive alternative to BSA. Nevertheless, engineering a different type of bead support or ZrO_2_-based trap column material which avoided the necessity for blocking agents would significantly improve the workflow in the future. Furthermore, and more generally, the optimized extended LC setup, equipped with an additional pump, could be utilized to integrate other substrate traps or chromatographic/enzymatic columns into the conventional HDX-MS apparatus to enhance the flexibility in HDX-MS analysis of complex protein samples.

Finally, we conducted measurements of MSP nanodiscs to determine the workability of the system. The number of identified peptides of MSP1E3D1 and the sequence coverage map—two crucial metrics in HDX-MS—have demonstrated equal performance compared to control measurements of the free protein, proving the functionality of the developed delipidation setup. Furthermore, we obtained 82.7% sequence coverage of the AcrB membrane protein solubilized in POPC nanodisc, demonstrating the applicability of the system on proteins of interest. This workflow will facilitate membrane protein characterization in HDX-MS to progress our understanding of protein dynamics in lipid environments. Overall, our developments will advance the field of membrane protein structural mass spectrometry, which is now at the point where a lipid milieu must be considered due to its putative relationship with protein structure and function.

## Supporting Information Available

Additional experimental details, materials, and methods, including experimental setup are provided in the supporting information. Supporting Figures are provided as PDF covering the following content: Automated delipidation setup; MSP1E3D1 lipid nanodisc preparation; AcrB nanodisc preparation; base peak ion chromatogram of AcrB in POPC nanodisc; detector calibration for delipidation experiments; overview of delipidation performance determined for EPL and Fos-choline-12; mass spectra of EPL; strategies for lipid elution from the lipid removal column; optimization of non-specific protein adsorption to ZrO_2_; protein measurements performed on the delipidation system (ZrO_2_ column) over time; evaluation of back-exchange and peptide carry-over; comparison of sequence coverage map from MSP1E3D1; base peak ion chromatogram of free MSP1E3D1, EPL, and POPC nanodisc at 0 sec labeling; stacked spectral plot of an MSP1E3D1 peptide measured in standard two-valve and extended three-valve configuration; HX-Express analysis of the peptide WDNLEKETEGLRQEMSKD (PDF)
Supporting Tables are provided in Excel format covering the following content: Overview of standard LC settings for lipid measurements; overview of LC settings to wash the system after lipid measurements; overview of standard LC settings for protein (PhosB and MSP1E3D1) measurements; overview of standard LC settings for AcrB nanodisc measurements; overview of LC settings to wash the system after protein measurements; overview of POPC removal rates of the delipidation system; overview of EPL removal rates of the delipidation system; overview of Fos-choline-12 removal rates of the delipidation system; evaluation of back-exchange; and evaluation of peptide carry-over (XLSX)
