# Supplementary material for: Chromatographic Phospholipid Trapping for Automated H/D Exchange Mass Spectrometry of Membrane Protein–Lipid Assemblies
Source: Anal Chem. 2023 Jan 27;95(5):3002–11. doi: 10.1021/acs.analchem.2c04876 (PMC9909672; doi:10.1021/acs.analchem.2c04876)
Supplement: Supplementary file 1 — ac2c04876_si_001.pdf [file ac2c04876_si_001.pdf]

Supporting Information

Chromatographic phospholipid trapping for automated H/D exchange mass spectrometry of membrane protein-lipid assemblies

Dietmar Hammerschmid[1,*], Valeria Calvaresi[1], Chloe Bailey[1], Benjamin Russell Lewis[1], Argyris Politis[1], Michael Morris[2], Laetitia Denbigh[2], Malcolm Anderson[2], Eamonn Reading[1,*]

[1] Department of Chemistry, King's College London, 7 Trinity Street, SE1 1DB London, UK

[2] Waters Corporation, Stamford Avenue, Altrincham Road, SK9 4AX Wilmslow, UK

Table of contents

Figure S1: Automated delipidation setup S5

Figure S2: MSP1E3D1 lipid nanodisc preparation S6

Figure S3: AcrB nanodisc preparation S7

Figure S4: Base peak ion chromatogram of AcrB in POPC nanodiscs S8

Figure S5: Detector calibration for delipidation experiments S9

Figure S6: Overview of delipidation performance determined for EPL and Fos-choline-12 S10

Figure S7: Mass spectra of EPL S11

Figure S8: Strategies for lipid elution from the lipid removal column S12

Figure S9: Optimization of non-specific protein adsorption to ZrO_2 S13

Figure S10: Protein measurements performed on the delipidation system (ZrO_2 column) over time S14

Figure S11: Evaluation of back-exchange and peptide carry-over S15

Figure S12: Comparison of sequence coverage map from MSP1E3D1 S16

Figure S13: Base peak ion chromatogram of free MSP1E3D1, EPL, and POPC nanodiscs at 0 sec labeling. S17

Figure S14: Stacked spectral plot of an MSP1E3D1 peptide measured in standard two-valve and extended three-valve configuration. S18

Figure S15: HX-Express analysis of the peptide WDNLEKETEGLRQEMSKD S19

Table S1: Overview of standard LC settings for lipid measurements S20

Table S2: Overview of LC settings to wash the system after lipid measurements S20

Table S3: Overview of standard LC settings for protein (PhosB and MSP1E3D1) measurements S20

Table S4: Overview of standard LC settings for AcrB nanodisc measurements S20

Table S5: Overview of LC settings to wash the system after protein measurements S20

Table S6: Overview of POPC removal rates of the delipidation system S20

Table S7: Overview of EPL removal rates of the delipidation system S20

Table S8: Overview of Fos-choline-12 removal rates of the delipidation system S20

Table S9: Evaluation of back-exchange S20

Table S10: Evaluation of peptide carry-over S20

Materials. Zirconia (ZrO_2) coated silica bulk (Cat No. 5425-U) was purchased from Supelco. Titansphere (TiO_2) 100Å 5µm, bulk, (Cat No. GL-5020-75000) was purchased from GL Sciences. 1-palmitoyl-2-oleoyl-sn-glycero-3-phosphocholine (POPC; Cat No. 850457) and E. coli Total Lipid Extract (EPL; Cat No. 100500) were purchased from Avanti Polar Lipids. N-Dodecylphosphocholine (Fos-choline-12; Cat No. F308S) and n-Dodecyl-β-D-Maltopyranoside (DDM; Cat No. D310) were purchased from Anatrace. Phosphorylase b (PhosB) from rabbit muscle (Cat No. P6635), Bovine Serum Albumin (BSA; Cat No. A2153), Deuterium oxide (99.9 atom%D; Cat No. 151882), Ammonium hydrox-ide (Cat No. 221228), 2,5-Dihydroxybenzoic acid (Cat No. 149357), and DL-Lactic acid (Cat No. 69785) were purchased from Sigma-Aldrich. Water (OptimaTM LC/MS grade; Cat No. W61), Acetonitrile (OptimaTM LC/MS grade; Cat No. A9551), Methanol (OptimaTM LC/MS grade; Cat No. A4561), Isopropanol (OptimaTM LC/MS grade; Cat No. A4611), and Formic acid (99.0+%, OptimaTM LC/MS grade; Cat No. A11750) were purchased from Fisher Scientific. Guanidinium hydrochloride (Cat No. 0118) and glycine (Cat No. 1504) were purchased from VWR Life Sciences. Potassium phosphate monobasic (Cat No. 094578) and dibasic (Cat No. 094672) were purchased from Flourochem.

Lipid Preparation. POPC and EPL (composition: 67.0% phosphatidylethanolamine, 23.2% phosphatidylglycerol, 9.8% cardiolipin) lipids were dissolved in cyclohexane and transferred into a glass vial. Cyclohexane was evaporated under a gentle N_2 stream. The dried lipid film was flash frozen in liquid N_2 and further freeze-dried for five hours. Lipids were resuspended (5 mg/mL) in 10 mM potassium phosphate buffer (pH 7.0) under gentle agitation for 1 hour, followed by sonication for 1 hour. Fos-choline-12 (5 mg/mL) was solubilized in solvent A. Before injection, lipids were diluted to the appropriate concentration and finally added to ice-cold 100 mM potassium phosphate buffer, pH 2.3 (1:1 vol/vol; final pH 2.5).

Preparation of MSP1E3D1 scaffold protein. pMSP1E3D1 containing "extended" MSP1D1 (Addgene) - which contains repeats of helices 4, 5 and 6, an N-terminal 7-his tag followed by spacer sequence and TEV protease cleavage site – was overexpressed in E. coli BL21(DE3) cells as described previously[1,2]. Cells were resuspended in lysis buffer (20 mM Na-phosphate, pH 8.0, 1% Triton X-100, 10 µg/ml DNAase, 1 mM PMSF, and a protease inhibitor tablet) and sonicated on ice applying 3 x 10 second pulses with 30 seconds breaks. Cell lysate was centrifuged at 25,000 x g for 30 minutes. The supernatant was mixed with 4 ml of Ni-NTA Superaffinity resin and incubated for 1 hour at room temperature under gentle agitation. The resin was loaded into a pre-chilled Biorad polyprep column for subsequent purification at 4 °C. The resin was washed with 10 column volumes (CVs) of wash buffer A (40 mM Tris-HCl, 300 mM NaCl, 1% Triton X-100, pH 8.0), 10 CVs of wash buffer B (40 mM Tris-HCl, 300 mM NaCl, 50 mM Na-cholate,

20 mM Imidazole, pH 8.0), and finally 10 CVs of wash buffer C (40 mM Tris-HCl, 300 mM NaCl, 50 mM Imidazole, pH 8.0). MSP1E3D1 protein was eluted with 5 CVs of elution buffer (40 mM Tris-HCl,

300 mM NaCl, 500 mM Imidazole, pH 8.0). Fractions were tested for purity by SDS-PAGE and the cleanest samples were pooled and dialyzed against MSP buffer (20 mM Tris-HCl, 100 mM NaCl, 0.5 mM EDTA, pH 7.4) at 4 °C. Finally, the sample was filtered using a 0.22 µm membrane, aliquoted, flash frozen with liquid nitrogen and stored at –80 °C.

Nanodisc Preparation. Lipid nanodiscs (POPC or EPL) were prepared as previously described[1,3]. Lipids were re-solubilized with MSP buffer (20 mM Tris-HCl, 100 mM NaCl, 0.5 mM EDTA, pH 7.4) containing 200 mM and 500 mM Na-cholate for POPC and EPL lipids respectively. MSP1E3D1 was added to the resuspended lipids at a 1:85 and 1:60 MSP:lipid molar ratio for POPC and EPL lipids respectively. Nanodisc mixtures with lipids, Na-cholate, and MSP were incubated at 4 °C for 30 min. BioBeads SM-2 (Bio-Rad) were added (~0.5 g of beads per 1 mL volume) to remove Na-cholate and drive nanodisc self-assembly. The MSP:lipid:cholate reconstitution was incubated with beads for at least 8 h with at least three bead changes. Beads were removed by filtration and generated nanodiscs were then purified using a Superdex 200 10/300 Increase GL column (GE Healthcare) in MSP buffer (**Figure S2**). Purity and size were assessed by SDS-PAGE and dynamic light scattering (DLS) using a Particle Size Analyzer LiteSizer 500 (Anton Parr).

Preparation of AcrB in Nanodiscs. AcrB was purified in DDM as described previously[4]. After purification, AcrB was inserted into nanodiscs according to the previously established protocols[1,5]. Briefly, AcrB in 0.03% (w/v) DDM detergent was mixed with POPC and MSP solution at a final 40:1:0.5 lipid:MSP1E3D1:AcrB molar ratio in MSP buffer with final concentration of 0.0116% (w/v) DDM and 16 mM Na-cholate. DDM was removed by the addition of SM2 Bio-beads (Bio-Rad) into the mixture and left in an orbital shaker overnight at 4 °C. AcrB nanodiscs were purified using a Superdex 200 Increase 10/300 (GE Healthcare) in AcrB sample buffer (50 mM sodium phosphate, 150 mM NaCl, 10% glycerol, pH 7.4) (**Figure S3**).

---

(1) Denisov, I. G.; Grinkova, Y. V.; Lazarides, A. A.; Sligar, S. G. J. Am. Chem. Soc. 2004, 126, 3477–3487.
(2) Denisov, I. G.; Baas, B. J.; Grinkova, Y. V.; Sligar, S. G. J. Biol. Chem. 2007, 282, 7066–7076.
(3) Roos, C.; Zocher, M.; Muller, D.; Munch, D.; Schneider, T.; Sahl, H-G.; Scholz, F.; Wachtveitl, J.; Ma, Y.; Proverbio, D.; Henrich, E.; Dotsch, V.; Bernhard, F. Biochim. Biophys. Acta Biomembr. 2012, 1818, 3098–3106.
(4) Reading, E.; Ahdash, Z.; Fais, C.; Ricci, V.; Wang-Kan, X.; Grimsey, E.; Stone, J.; Malloci, G.; Lau, A. M. Findlay, H.; Konijnenberg, A.; Booth, P. J. Ruggerone, P.; Vargiu, A. V.; Piddock, L. J. V.; Politis, A. Nat. Commun. 2020, 11, 5565.
(5) Daury, L.; Orange, F.; Taveau, J.; Verchere, A.; Monlezun, L.; Gounou, C.; Marreddy, R. K. R.; Picard, M.; Broutin, I.; Pos, K. M.; Lambert, O. *Nat. Commun.* **2016**, 7, 10731.

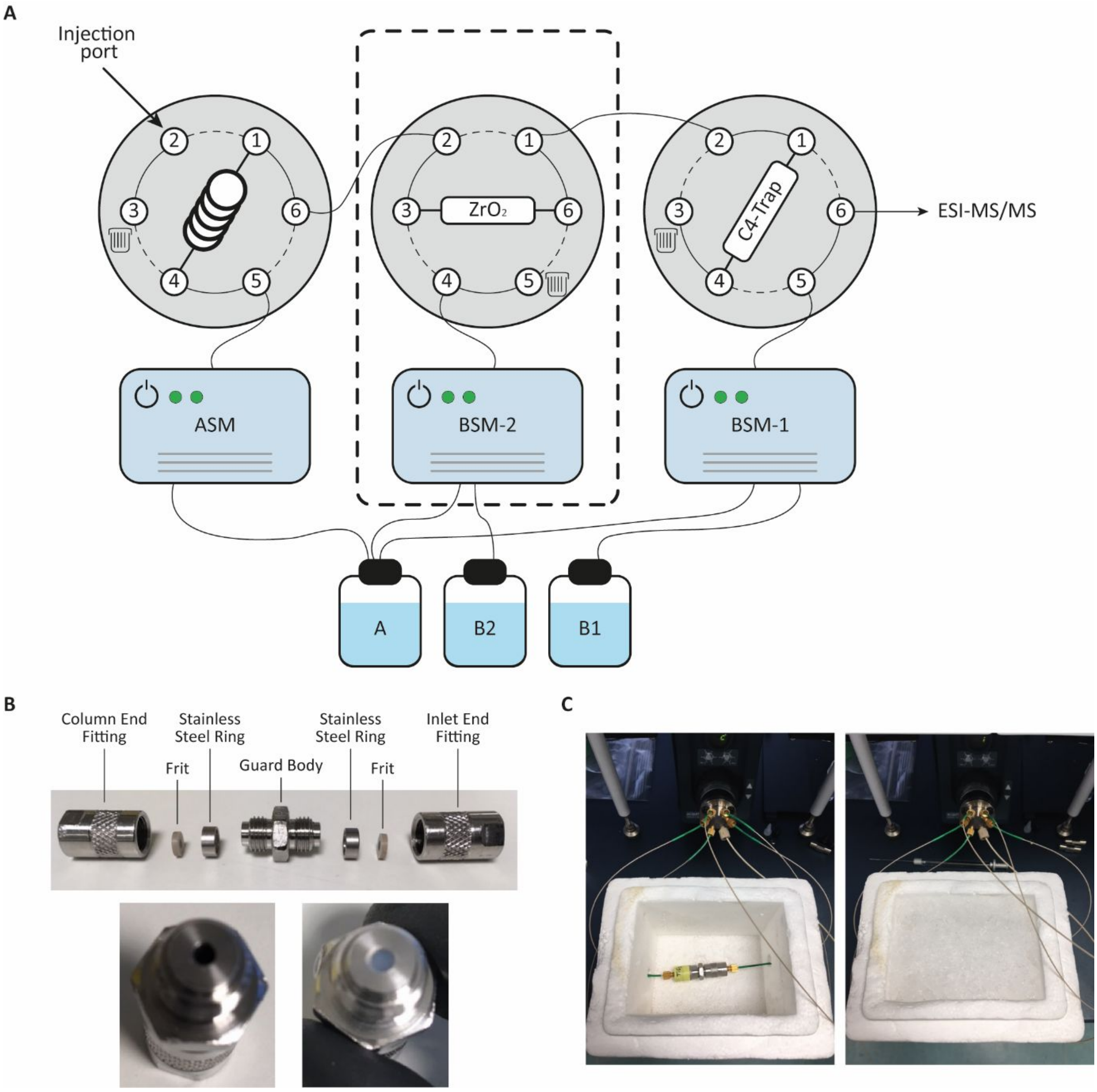

Figure S1: Automated delipidation setup. (A) Flow path and column configuration applied for phospholipid measurements to determine the delipidation efficiency. (B) Components of the lipid removal column including images from before and after column packing. (C) Image of ice box to keep the lipid removal column at 0 °C during experiments.

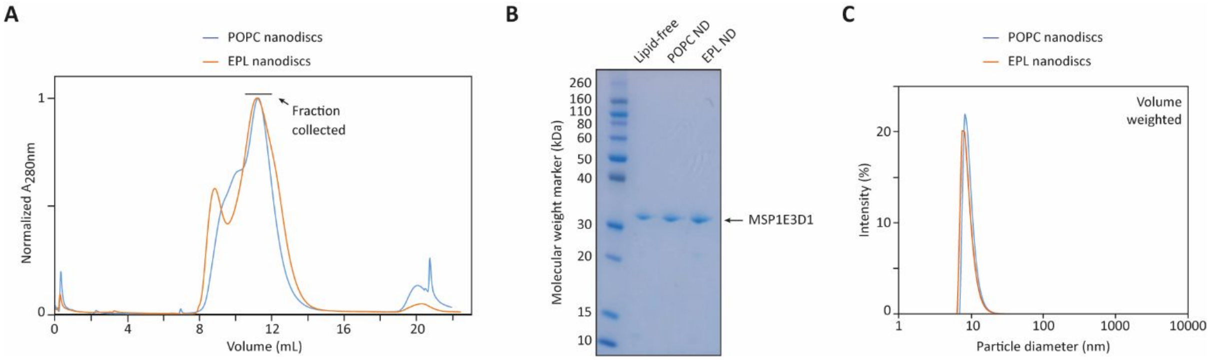

Figure S2: MSP1E3D1 lipid nanodisc preparation. (A) Traces of size exclusion chromatography (Superdex 200 10/300 Increase GL) of POPC and EPL MSP1E3D1 lipid nanodiscs. (B) SDS-PAGE analysis of lipid-free as well as POPC and EPL nanodisc MSP1E3D1 (predicted molecular weight for amino acid sequence: 32,599.98 Da). (C) Traces of dynamic light scattering of POPC and EPL nanodiscs.

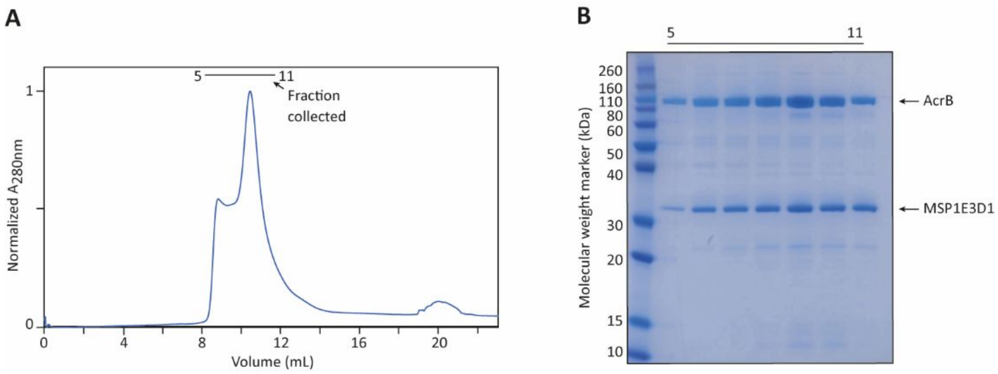

Figure S3: AcrB nanodisc preparation. (A) Traces of size exclusion chromatography (Superdex 200 10/300 Increase GL) of AcrB in POPC lipid nanodiscs. (B) SDS-PAGE analysis of different SEC fractions collected (the predicted molecular of MSP1E3D1 and AcrB is 32,599.98 Da and 114,450.58 Da, respectively).

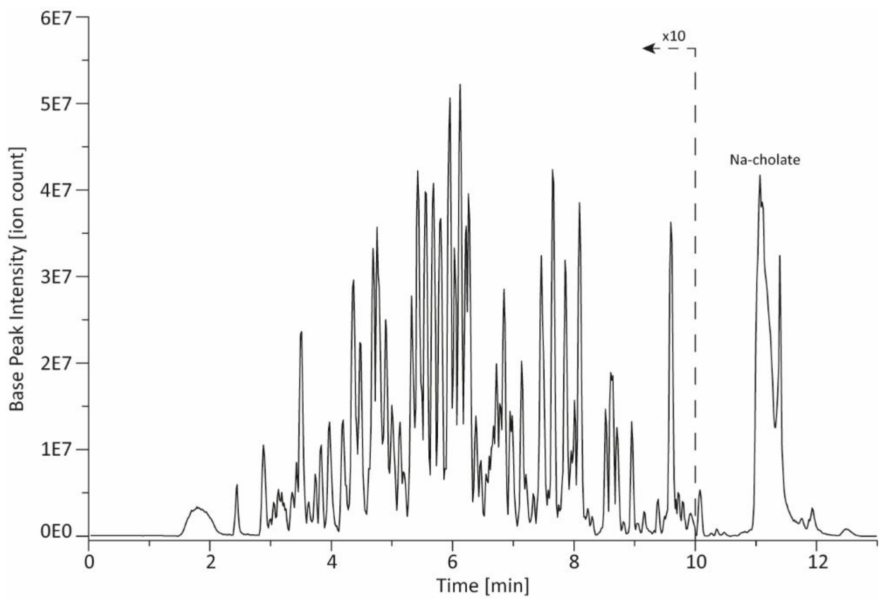

Figure S4: Base peak ion chromatogram of AcrB in POPC nanodiscs. Area between 3- and 10-minutes highlights elution of vast number of peptides.

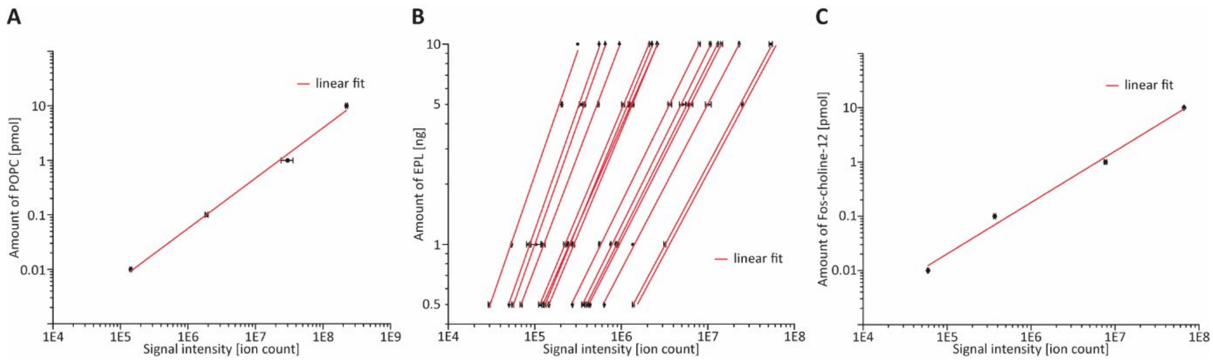

Figure S5: Detector calibration for delipidation experiments. (A) Linear fit of POPC amount versus signal intensity. (B) Linear fit of EPL amount versus signal intensity of the different lipids in the mixture. (C) Linear fit of Fos-choline-12 amount versus signal intensity. Information on slope and intercept of the different calibration curves can be found in Table S6 (A), Table S7 (B), and Table S8 (C). The formula y = 10^((slope * log(x)) + intercept) was used to calculate the remaining amount of lipid after delipidation (x = lipid signal).

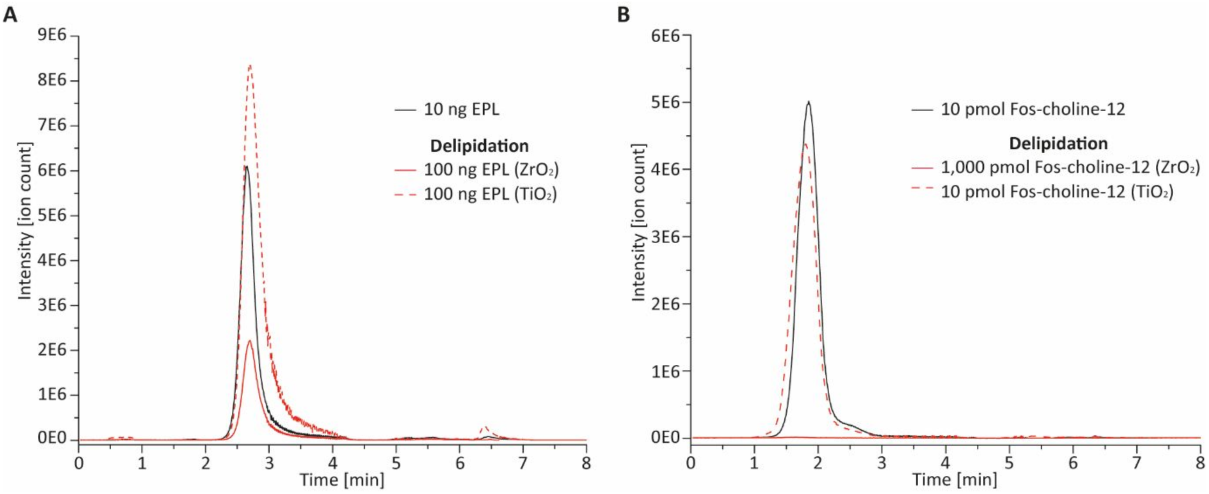

Figure S6: Overview of delipidation performance determined for EPL and Fos-choline-12. (A) Extracted ion chromatogram (EIC) of phosphoethanolamine 33:1 (704.55 m/z) before and after delipidation. (B) EIC of Fos-choline-12 (352.26 m/z) before and after delipidation. Black and red traces (ZrO_2: solid, TiO_2: dashed) correspond to measurements before and after sample delipidation respectively.

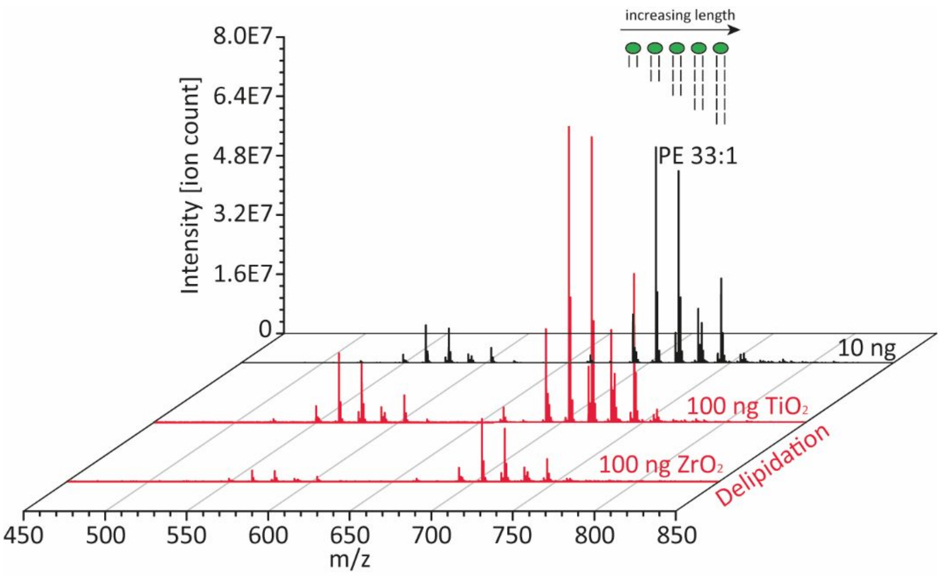

Figure S7: Mass spectra of EPL. Comparison of mass spectra of EPL before and after delipidation with a ZrO_2-packed lipid removal column.

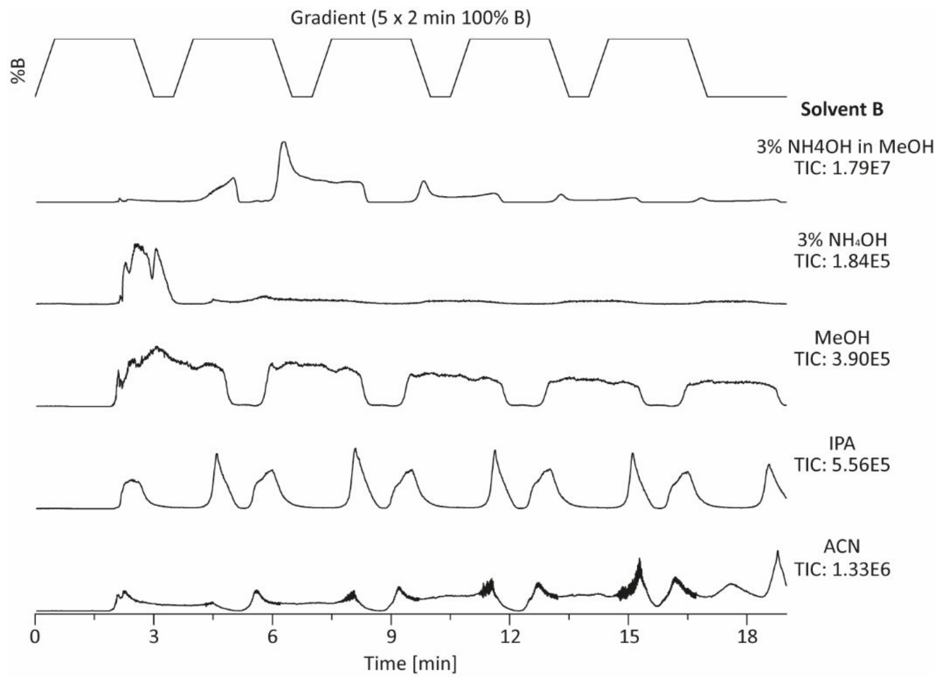

Figure S8: Strategies for lipid elution from the lipid removal column. EIC of POPC after applying a gradient of 5 x 2 minutes 100% solvent B, e.g. acetonitrile, isopropanol, methanol, and 3% ammonium hydroxide in H_2O or methanol, to the lipid removal column.

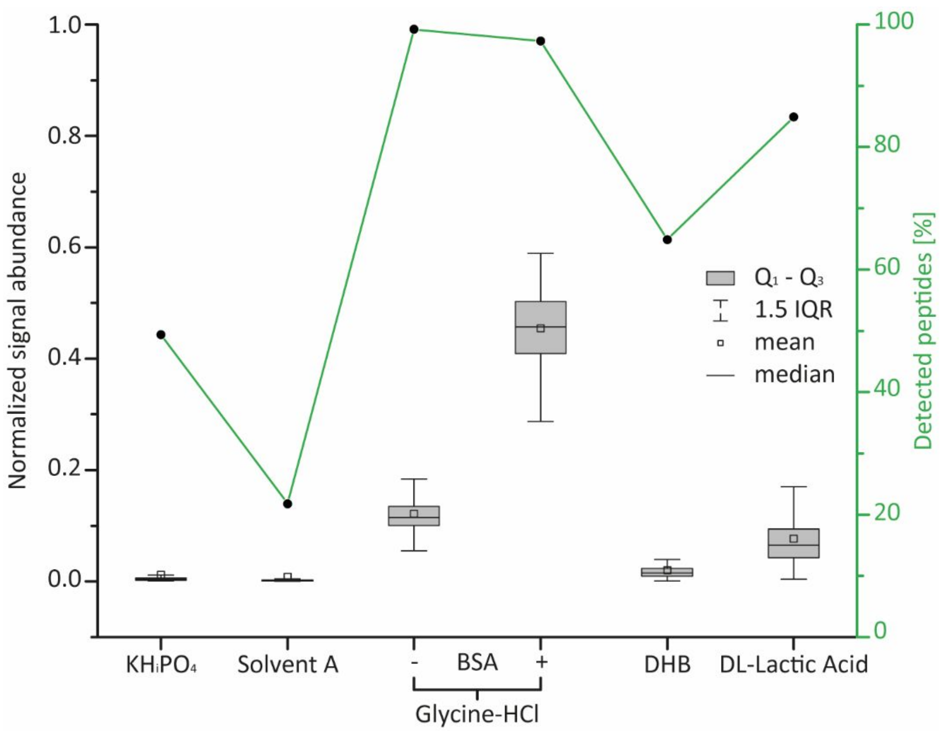

Figure S9: Optimization of non-specific protein adsorption to ZrO_2. Boxplot of normalized peptide signal intensities obtained from the delipidation system relative compared to the standard two-valve HDX-MS configuration. Different quench buffers, i.e. 100 mM potassium phosphate pH 2.3, solvent A (0.23% formic acid), 500 mM glycine-HCl pH 2.35, 5 mg/mL DHB in H_2O, and 15 mg/mL DL-lactic acid in H_2O, were tested to potentially shield non-specific protein binding. Glycine-HCl was performed on both a BSA blocked and unblocked lipid removal column. The right y-axis indicates the percentage of peptides which could be identified through a suitable signal-to-noise ratio.

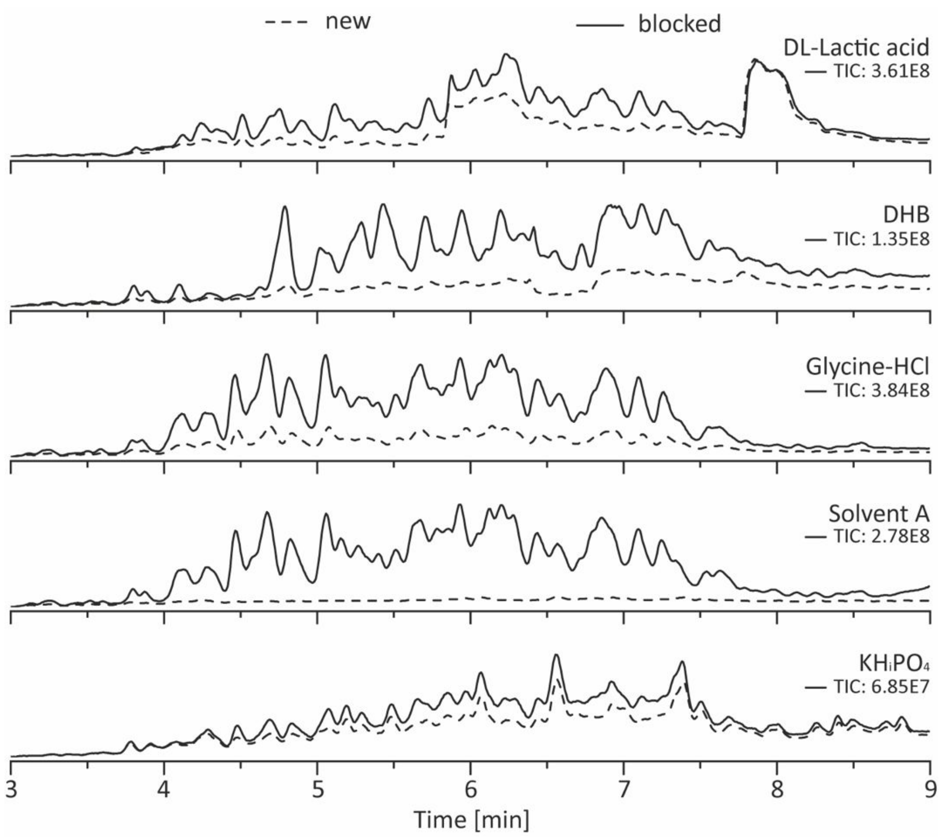

Figure S10: Protein measurements performed on the delipidation system (ZrO_2 column) over time. PhosB measurements after applying different quench buffers, i.e. 100 mM potassium phosphate pH 2.3, solvent A (0.23% formic acid), 500 mM glycine-HCl pH 2.35, 5 mg/mL DHB in H_2O, and 15 mg/mL DL-lactic acid in H_2O, on a new (dashed lines) and blocked (solid lines) ZrO_2 lipid removal column.

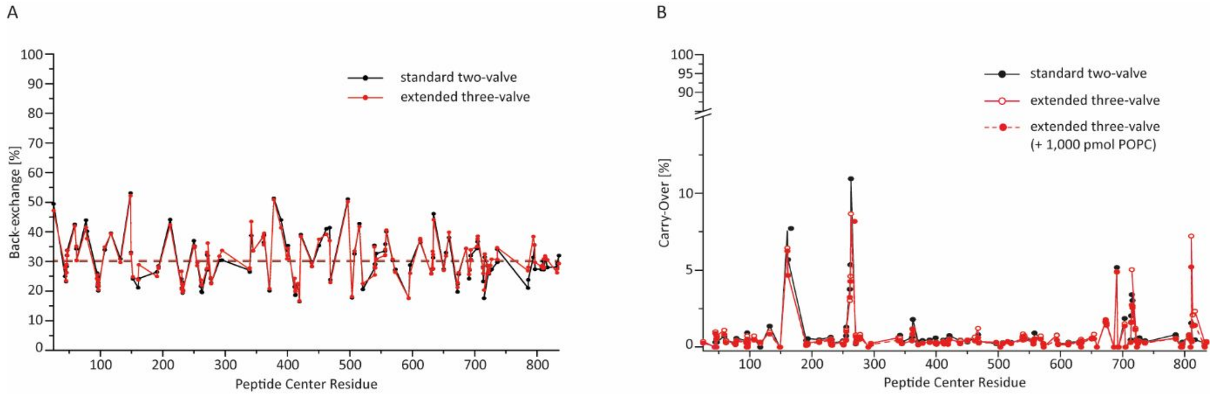

Figure S11: Evaluation of back-exchange and peptide carry-over. (A) Level of back-exchange compared between standard two-valve (solid black) and extended three-valve (solid red) configuration. Dashed lines indicate the average of back-exchange. (B) Comparison of peptide carry-over between standard (solid black) and delipidation system with (dashed red) and without (solid red) 50 x molar excess of POPC.

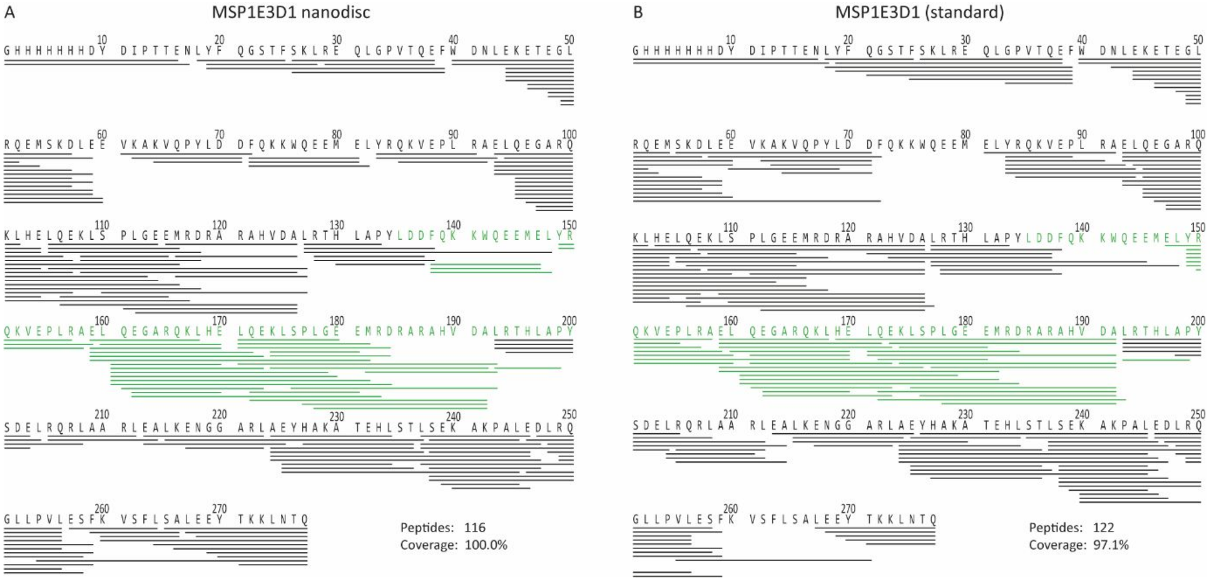

Figure S12: Comparison of sequence coverage map from MSP1E3D1. (A) Sequence coverage map of MSP1E3D1 from nanodisc form obtained on three-valve HDX-MS configuration. (B) Sequence coverage map of free MSP1E3D1 obtained on two-valve HDX-MS configuration. The sequence in green highlights the repeat of helices 4, 5, and 6.

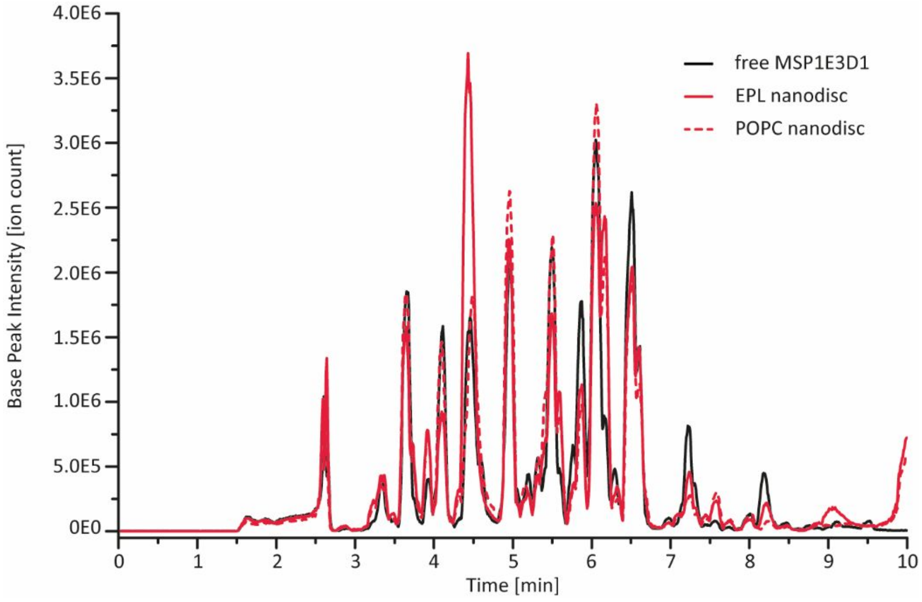

Figure S13: Base peak ion chromatogram of free MSP1E3D1, EPL, and POPC nanodiscs at 0 sec labeling. The chromatograms origin from different datasets acquired on subsequent days using the same BSA blocked ZrO_2 phospholipid trap column. All chromatograms are of comparable intensity, indicating that depletion of BSA is minimal.

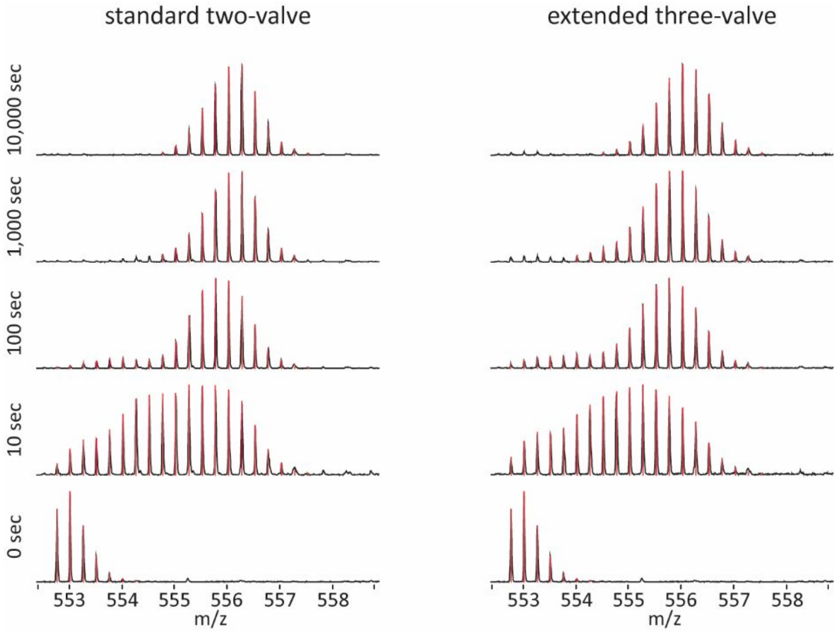

Figure S14: Stacked spectral plot of an MSP1E3D1 peptide measured in standard two-valve and extended three-valve configuration. The selected peptide (residues 40-57; m/z 552.76; +4) displays no differences across the different configurations applied.

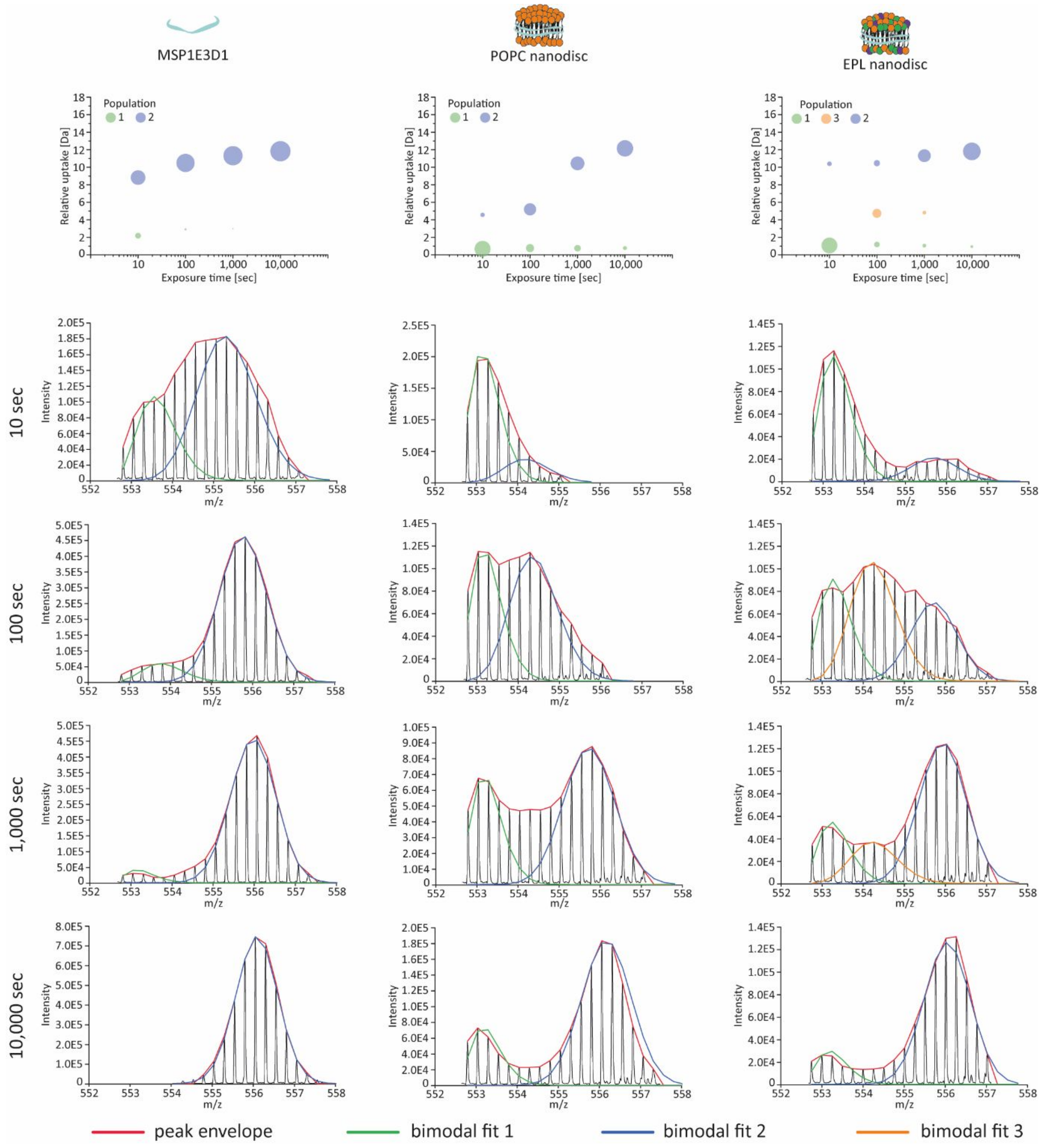

Figure S15: HX-Express analysis of the peptide WDNLEKETEGLRQEMSKD. Bimodal fitting of the different spectra after deuteration. The bubble plots (top) show the deuterium uptake of each population individually. The size of the bubbles indicates the relative abundance of the different populations to each other.

Table S1: Overview of standard LC settings for lipid measurements. BSM-2 was only used in extended three-valve configuration.

Table S2: Overview of LC settings to wash the system after lipid measurements. BSM-2 was only used in extended three-valve configuration.

Table S3: Overview of standard LC settings for protein (PhosB and MSP1E3D1) measurements. BSM-2 was only used in extended three-valve configuration.

Table S4: Overview of standard LC settings for AcrB nanodisc measurements.

Table S5: Overview of LC settings to wash the system after protein measurements. BSM-2 was only used in extended three-valve configuration.

Table S6: Overview of POPC removal rates of the delipidation system. Delipidation efficiency was determined for different column dimensions and for manual lipid removal based on ZrO_2 beads.

Table S7: Overview of EPL removal rates of the delipidation system. Detailed analysis for single lipids in EPL.

Table S8: Overview of Fos-choline-12 removal rates of the delipidation system.

Table S9: Evaluation of back-exchange. Level of back-exchange was determined for both the standard two-valve and the extended three-valve configuration using PhosB.

Table S10: Evaluation of peptide carry-over. Peptide carry-over was determined for both standard two-valve and extended three-valve configuration using PhosB. Furthermore, the impact of lipids on carry-over of the delipidation system (three-valve) was assessed by adding 1,000 pmol POPC (50 x molar excess) to PhosB.
